# Secretion of Extracellular Vesicles Into the Mesenteric Lymph During Fasting and Lipid Absorption

**DOI:** 10.1002/jex2.70170

**Published:** 2026-07-23

**Authors:** Tianyu Hang, Rita Wang, Kundanika Mukherjee, Uday Sandhu, Fengxia Xiao, Dylan Burger, Changting Xiao

**Affiliations:** ^1^ Department of Anatomy, Physiology and Pharmacology, College of Medicine University of Saskatchewan Saskatoon Saskatchewan Canada; ^2^ Kidney Research Centre Ottawa Hospital Research Institute Ottawa Ontario Canada; ^3^ Department of Cellular and Molecular Medicine, Faculty of Medicine University of Ottawa Ottawa Ontario Canada

## Abstract

The intestine plays critical roles in nutrient homeostasis and systemic health. The small intestine is the major organ that packages dietary lipids into chylomicrons and secretes them into the mesenteric lymph. Extracellular vesicles (EVs) as a mechanism for cell‐to‐cell communication often exhibit organ specific characteristics. EV secretion from the intestine into the mesenteric lymph—a unique biofluid—remains unexplored. To characterize gut‐derived EVs, Sprague‐Dawley rats were surgically implanted with cannula in the mesenteric lymph duct (for lymph collection) and the duodenum (for lipid infusion). Lymph fluid was collected for assessment of EV secretion. EVs in lymph fluids collected before and after lipid infusion were characterized by transmission electron microscopy, nanoparticle tracking analysis, and further analyzed by flow cytometry with antibodies against CD63, CD81, CD9 and apolipoprotein B (ApoB). Lipid infusion increased lymph triglyceride output peaking at 2 h. Lymph fluids contained EVs with diameters in the range of 20 to 300 nm and the signals of specific EV markers CD63, CD81 and CD9 were significantly elevated following lipid infusion. Depletion of chylomicrons from lymph had differential effects on the percentage and intensity of CD63, CD81 and CD9. In addition, these EVs exhibited distinct patterns in ApoB^+^ particle population, with the median fluorescence intensity of CD63 and CD81 being significantly higher than that of CD9. To conclude, EVs secreted into the mesenteric lymph could rapidly respond to lipid supply. EVs may bind to and co‐secrete with chylomicrons. Finally, gut‐derived EVs during active lipid absorption may include multiple subtypes with different affinities to chylomicrons. Collectively, these findings highlight the intestine as an EV secretion organ and the mesenteric lymph as an important biofluid harboring EVs, which opens a new venue for future investigation of biological functions of gut‐derived EVs.

## 1 Introduction

The gut plays important roles in health and disease. As the primary organ for digestion and absorption of dietary nutrients, especially dietary fats, it helps maintain whole‐body energy homeostasis. The gut epithelium serves as a barrier between the inner body and the outside environment, with the gut lumen housing diverse microbiota that mediates host health. The gut also secretes many gut hormones that regulate systemic physiological functions. Notably, dysregulated gut functions are linked to various diseases, including chronic metabolic diseases, such as type 2 diabetes and obesity (Wit et al. 2022). Identifying gut‐derived factors have yielded novel therapeutics for diseases, exemplified by the recent development of gut hormone‐based therapies for type 2 diabetes and obesity (Drucker 2025).

Extracellular vesicles (EVs) are particles of varied sizes and content released from cells, delimited by a lipid bilayer, and cannot replicate on their own (Welsh et al. 2024, Doyle and Wang 2019, Raposo and Stoorvogel 2013). EVs are classified into exosomes, microvesicles, and apoptopic bodies based on their mechanism of biogenesis. EVs can also be classified into small EVs (<200 nm in diameter) and large EVs (>200 nm in diameter) based on their physical properties. As complete separation of EV sub‐populations and confirmation of the origin of EV isolates is challenging, the use of the inclusive term “EV” is preferred to biogenesis‐based terms and will be employed throughout this manuscript (Welsh et al. 2024). Exosomes, often the dominant subpopulation of small EVs (<200 nm in diameter), are formed inside cells through an endosomal sorting complexes required for transport (ESCRT) pathway and an ESCRT‐independent pathway that depends on sphingomyelinase and tetraspanin family proteins, including CD63, CD9, and CD81 (Doyle and Wang 2019). EVs contain cell‐specific surface markers and various cargo content, for example, proteins, lipids, and nucleic acids derived from their originating cells. EVs represent an important cell‐to‐cell communication mechanism in regulating the functions of proximal and distal target cells and play important roles in the pathogenesis of diseases. As such, EVs bear promises as biomarkers and novel therapeutics of various diseases, for example, cancer, type 2 diabetes, kidney diseases, and atherosclerosis (Dini et al. 2020, Cione et al. 2021, Jafari et al. 2022, Martínez and Andriantsitohaina 2017, Salomon et al. 2022, Erdbrugger et al. 2023). Studies on EV secretion from various organs/tissues, for example, liver, adipose, and muscle, have demonstrated specific biological functions of EVs from such sources (Miotto et al. 2024, Liu et al. 2025, Haque et al. 2025). The intestine as an EV secreting organ has not been investigated.

Following secretion from cells into extracellular space, EVs are transported in biofluids. To date, studies have focused on EVs from several biofluids, namely blood, urine, and breast milk (Luo et al. 2023, Medel‐Martinez et al. 2024, Leung et al. 2025). The lymphatic vasculature is an important conduit of fluids circulating throughout the body, carrying diverse biological entities, such as proteins, cytokines, and immune cells. Importantly, the lymphatic system is a metastasis route for cancer. It has been found that lymph fluid in the proximity of breast tumour is enriched with tumour markers in humans (Ekström et al. 2022) and rodents (Miao et al. 2025). Cancer‐derived EVs mediate lymphatic remodelling in mice (García‐Silva et al. 2021, Leary et al. 2022). The presence of EVs in lymph fluid collected from the thoracic duct in mice suggested potential relationships among EV, atherosclerosis, and lymphatic dysfunction (Milasan et al. 2016). However, in large part due to the lack of approaches for fluid collection, EVs in lymph fluids in general, and in lymph fluids of specific organ drainage have not been well studied.

The mesenteric lymph duct drains intestinal secretions specifically. During dietary fat absorption, lipids are packaged into chylomicrons in intestinal absorptive cells (enterocytes). Chylomicrons are lipoprotein particles, composed of a neutral lipid core of triglycerides and cholesterols, surrounded by a phospholipid monolayer, and carry the structural protein apolipoprotein B‐48 (ApoB‐48) on its membrane. Following secretion from enterocytes, chylomicrons are transported in the mesenteric lymph duct before joining the blood circulation. The mesenteric lymph also contains non‐lipid molecules, such as the gut hormones glucagon‐like peptide‐1 (D'Alessio et al. 2007, Ohlsson et al. 2014, Jejelava et al. 2018). It is highly likely that the mesenteric lymph drains EV secretion from the intestine, which has not been investigated.

Here we take advantage of a unique technique, that is mesenteric lymph duct cannulation, to obtain ‘liquid biopsy’ of the intestine and assess EV secretion from this organ in the rat. As the first study of this kind and a proof of concept, gut‐derived EVs were evaluated in rats before and after lipid delivery into the small intestinal lumen to gain insights into EV secretion both in the fasting condition and during an active physiological process (lipid absorption) specific to this organ.

## 2 Methods

### 2.1 Animals

Male Sprague‐Dawley rats (200–350 g) were purchased from Charles River Laboratories (Senneville, QC, Canada). The animals were housed in pairs in polycarbonate cages in a room with controlled temperature, humidity, and an automatic 12‐h light/12‐h dark cycle. They had free access to a standard laboratory diet (LabDiet, St. Louis, MO; Prolab RMH 3000; calories from protein 26.1%, fat 14.4%, and carbohydrates 59.5%) and water. Rats were acclimated to these conditions for at least 1 week prior to surgery. All animal procedures were approved by the Animal Research Ethics Board of the University of Saskatchewan.

### 2.2 Lipid or Saline Infusion

Following acclimation and an overnight fast, rats were surgically implanted with catheters into the duodenum (for lipid or saline infusion) and the mesenteric lymph duct (for lymph fluid collection), as described in our previous study (Stahel et al. 2019). Briefly, a polyvinyl chloride tubing (0.2 mm ID, 0.5 mm OD) was inserted into the mesenteric lymph duct, secured in place with a drop of tissue glue, and exteriorized at the right abdominal flank. A silicone tubing (0.5 mm ID, 0.8 mm OD) was inserted 2 cm into the duodenum through a fundal incision in the stomach and secured with a purse‐string suture. Subsequently, the rats were housed in Bollman restraint cages at a controlled ambient temperature of 26°C. Saline with 5% glucose was provided through the intraduodenal catheter at a rate of 3 mL/h for hydration. The rats recovered overnight while the liquid infusion was switched to saline alone to mimic an overnight fast. On the following day, the rats were randomly assigned to two treatment groups to receive a bolus infusion of either lipid (Intralipid 20%, 1.5 mL) or saline (1.5 mL) as control into the duodenum. Saline infusion was maintained throughout the study to keep the rats hydrated and to maintain mesenteric lymph duct catheter patency.

### 2.3 Lymph Fluid Collection and Cell‐Free Lymph Fluid Preparation

Lymph fluid was collected for one hour before lipid or saline infusion as baseline. Following lipid or saline infusion, lymph fluid was collected at hourly intervals for up to 4 h. All lymph collection tubes were placed on ice. Fresh lymph samples were centrifuged at 2,500 g for 10 min to remove cells and debris and stored at −80°C until further analysis.

### 2.4 EVs Isolation

EVs in cell‐free lymph were obtained in two steps, first with chylomicron depletion, then with EV enrichment, following a previously described protocol (Zhang et al. 2023).

#### 2.4.1 Chylomicrons Depletion

Cell‐free lymph (1 mL) was thawed and centrifuged using a Beckman XPN100 at 20,000 g for 20 mins twice to float chylomicrons (Fidge and McCullagh 1981). Following the first centrifugation, the middle and bottom fractions (800 µL) were carefully transferred to a new tube, added with 200 µL of PBS to restore the original volume, and underwent another centrifugation. At the end of the 2^nd^ centrifugation, the middle and bottom fractions (800 µL) were collected into a new tube and was referred to as the chylomicron‐free fraction. The top fractions (200 µL) from both centrifugations were combined and referred to as the chylomicron fraction. For flow cytometry, PBS was added to both fractions to restore the original volume.

#### 2.4.2 EV Enrichment

The chylomicron‐free fraction was centrifuged at 100,000 g for 90 mins twice to enrich EVs, using a Beckman XPN100 ultracentrifuge with a SW41 Ti rotor or a Beckman Optima MAX‐XP ultracentrifuge with a TLA20.1 rotor depending on sample volume. The supernatant was discarded, and the pellet was resuspended in PBS.

### 2.5 Analyses of Gut‐Derived EVs

EV isolates were characterized on the basis of size and protein composition. For size distributions, nanoparticle tracking analysis (NTA) was carried out using a ZetaView PMX110 system (Particle Metrix, Meerbusch, Germany) as described previously (Myette et al. 2023, Tschirhart et al. 2023, Myette et al. 2025). Samples were diluted into the system's range of detection using 1X PBS and particle size profiles were obtained using the following settings: 11 camera positions with a 2 s video length, a camera frame rate of 30 FPS and a temperature of 21°C. For detergent control, samples were incubated with 0.1% SDS for 30 mins before analysis. The system was calibrated using 105 and 500 nm polystyrene beads and ZetaView software (ver 8.02.28) was used for all analysis.

The protein composition of EV isolates was assessed by Western blot. Briefly, EV isolates were lysed in RIPA buffer containing proteinase and phosphatase inhibitors. Lysates were then denatured at 95°C for 5 min and 10 µg of protein lysates per sample were loaded onto SDS‐PAGE gels and separated by electrophoresis. Proteins were then transferred onto a polyvinylidene difluoride (PVDF) membrane blocked with 3% skim milk in Tris‐buffered saline and 0.1% Tween 20 (TBST) for 1 h at room temperature. The membrane was incubated with antibodies to a membrane‐associated protein commonly identified in EVs (Flotillin‐1, Abcam, Cat# 133497, 1:1000), a nuclear protein associated with cell compartments other than EVs (Nucleoporin p62, Santa Cruz, Cat# 48389, 1:2000), and a cytosolic protein with promiscuous incorporation into EVs (GAPDH, ThermoFisher, Cat# MA5‐15738, 1:2000) overnight at 4°C. After washing, membranes were incubated with appropriate secondary antibodies (BioRad, 1:2000) for 1 h at room temperature. Clarity Western ECL Substrate was used to visualize the protein bands.

#### 2.5.1 Flow Cytometry (FCM)

FCM was used to detect EVs and chylomicron particles, using fluorescence conjugated antibodies against CD63, CD9 and CD81 (EV markers for detecting or immunocapturing EVs (Campos‐Silva et al. 2019)) and ApoB(A‐6) (chylomicron marker) and using aldehyde/sulphate latex beads (4% w/v, 4 µm, Invitrogen Cat: A37304). Samples with beads alone were used as negative control. The latex bead working solution was prepared by diluting 5 µL of latex beads with 500 µL of PBS. 50 µL of latex bead working solution was added to 50 µL of cell‐free lymph fluid (before/after Intralipid infusion). In beads‐only control, 50 µL latex bead working solution was added to 50 µL of PBS. For detergent control, sample‐beads complex (100 µL) was pre‐incubated with the same volume of 0.1% Triton‐X for 30 min. Beads‐only control, detergent control and lymph samples were processed the same way following protocols. After a 15‐min incubation at room temperature, 900 µL of PBS was added and the samples were incubated for another 2 h on a platform rocker at room temperature. Samples were then centrifuged at 5,000 g for 5 mins, and the supernatant was discarded. The pellets were resuspended with 100 µL of FBS and incubated for 30 mins at room temperature. At the end of the incubation, 1 mL of PBS was added to wash the sample, followed by centrifugation at 5,000 g for 5 mins. Pellets were then resuspended in PBS. 5 µL of antibody solution containing anti‐CD63‐PE (Cat# sc‐5275 PE, Santa Cruz), anti‐CD9‐AF406 (Cat# sc‐13118 AF405, Santa Cruz) or anti‐81‐AF647 (Cat# sc‐166029 AF647, Santa Cruz) was added to 100 µL of samples and incubated for 30 mins on ice, with all samples protected from light. Each of the antibodies was incubated with samples separately. In another set of experiments, anti‐ApoB(A‐6)‐FITC (Cat# sc‐393636 FITC, Santa Cruz) was added during the incubation of anti‐CD63‐PE, anti‐CD9‐AF406, or anti‐CD 81‐AF647. Individual tubes with a single fluorochrome‐conjugated antibody were set up as single‐stained controls for performing spectral compensation. ABC anti‐mouse beads kit (Cat# A10344, Invitrogen) was used to demonstrate high expression (capture beads) and low expression (negative beads) for each antibody, following the manufacturer's protocol. After incubation, samples were washed by centrifugation at 5,000 g for 5 mins twice. The supernatants were discarded, and the pellets were resuspended in 100 µL PBS.

A Beckman CytoFLEX Flow Cytometer was used to analyze samples. Data Analysis was performed using FlowJo software. Forward scatter (FSC) against side scatter (SSC) was used to gate on beads population, and unknown population. To identify singlets and exclude doublets and higher‐order aggregates, forward scatter area (FSC‐A) against forward scatter height (FSC‐H) was used. The singlet gate was defined by selecting the population with a linear relationship between FSC‐A and FSC‐H and excluding events outside this region. All percentages refer to events within the singlet gate. Gating strategies (as shown in Figure S3 and Figure S7 with representative plots from a typical sample) were consistently applied across all samples in the experiment. During analysis, median fluorescence intensity (MFI) and positive percentage were used to evaluate differences between groups. The MFI and positive percentage of beads‐only control were used to gate CD63^+^, CD81^+^, CD9^+^ and ApoB^+^ events; <1% positive events were considered as a threshold. All results shown were the differences between samples and beads‐only control.

#### 2.5.2 Transmission Electron Microscopy (TEM)

EV morphology was observed with a Hitachi HT7700 transmission electron microscope. EVs were purified as above using lymph samples collected in the Intralipid group (*n* = 3) at −1 and 2 h after lipid load. Negative staining was used to process the samples. In brief, 400 Cu formvar/carbon grids were glow discharged to make the surface hydrophilic. Next, 5 uL of samples was placed on a grid for 2 mins. After removing excess solution with a filter paper, the grid was washed once by floating on a drop of distilled water on parafilm for 20 s. The grid was then quickly placed on a drop of 2% uranyl acetate for 1 min. The excess solution was removed with a filter paper, and the sample was air dried. Magnifications of 30x, 60x and 150x were used during observation.

### 2.6 Lymph Triglyceride Assay and Lymph Triglyceride Output Calculation

Triglyceride (TG) concentrations in lymph were analyzed using a commercial kit (FUJIFILM, Lexington, MA). Lymph flow rate was calculated as the lymph volume per hour for each time point. Lipid output was calculated as the product of lymph flow rate and TG concentration (Mukherjee and Xiao 2024).

### 2.7 Statistical Analysis

Two‐way ANOVA or mixed‐effects analysis followed by Tukey's multiple comparisons tests were used for statistical analyses. All experiments were done twice as technical replicate. A P value < 0.05 was considered as significance.

## 3 Results

### 3.1 Lipid Infusion Increases Lipid Secretion From the Intestine

**FIGURE 1:**
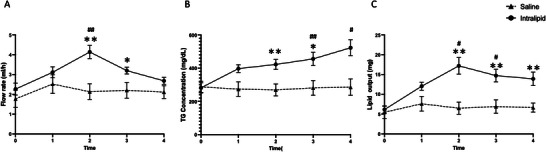
**Lipid infusion increases lymph triglyceride output**. (A) Lymph flow rate, (B) TG concentrations, and (C) TG output following intraduodenal Intralipid or saline infusion in Sprague‐Dawley rats. Two‐way ANOVA or mixed‐effects analysis with multiple comparisons followed by Tukey's multiple comparisons test was used for statistical analysis. Results are expressed as mean ± SEM: Saline, *n* = 5 rats; Intralipids, *n* = 6 rats. * *P* < 0.05 and ** *P* < 0.01, Saline vs Intralipid; # *P* < 0.05, ## *P* < 0.01, after Intralipid vs before Intralipid.

Lipid infusion directly into the duodenal lumen provides lipids to the intestine with precise timing. We administered Intralipid, a common lipid emulsion, into the duodenum to mimic dietary fat ingestion. As expected, and in line with previous studies in the same mesenteric lymph duct cannulated rat model (Mukherjee and Xiao 2024), lipid infusion increased lipid output compared with saline infusion (Figure 1). With saline infusion, lymph flow rate, TG concentration, and TG output remained comparable to baseline. In contrast, with lipid infusion, lymph flow rate began to rise and peaked at the second hour. Although lymph flow rate began to decline towards baseline after the second hour, it remained significantly higher than with saline infusion at the third hour (*P* < 0.05, Figure 1A). Lymph TG concentrations were elevated after lipid infusion (*P* < 0.05, Figure 1B). As a result, with lipid infusion, TG output was elevated from baseline, peaked at the 2^nd^ hour, and was significantly higher than with saline infusion at the 2^nd^ and 3^rd^ hour (*P* < 0.05, Figure 1C). Based on these results, we selected the 2^nd^ hour following lipid infusion as the timepoint for further analyses of lymph fluid alterations after lipid loading and used lymph collected before lipid infusion as the control.

### 3.2 The Intestine Secretes EVs Into Mesenteric Lymph

**FIGURE 2:**
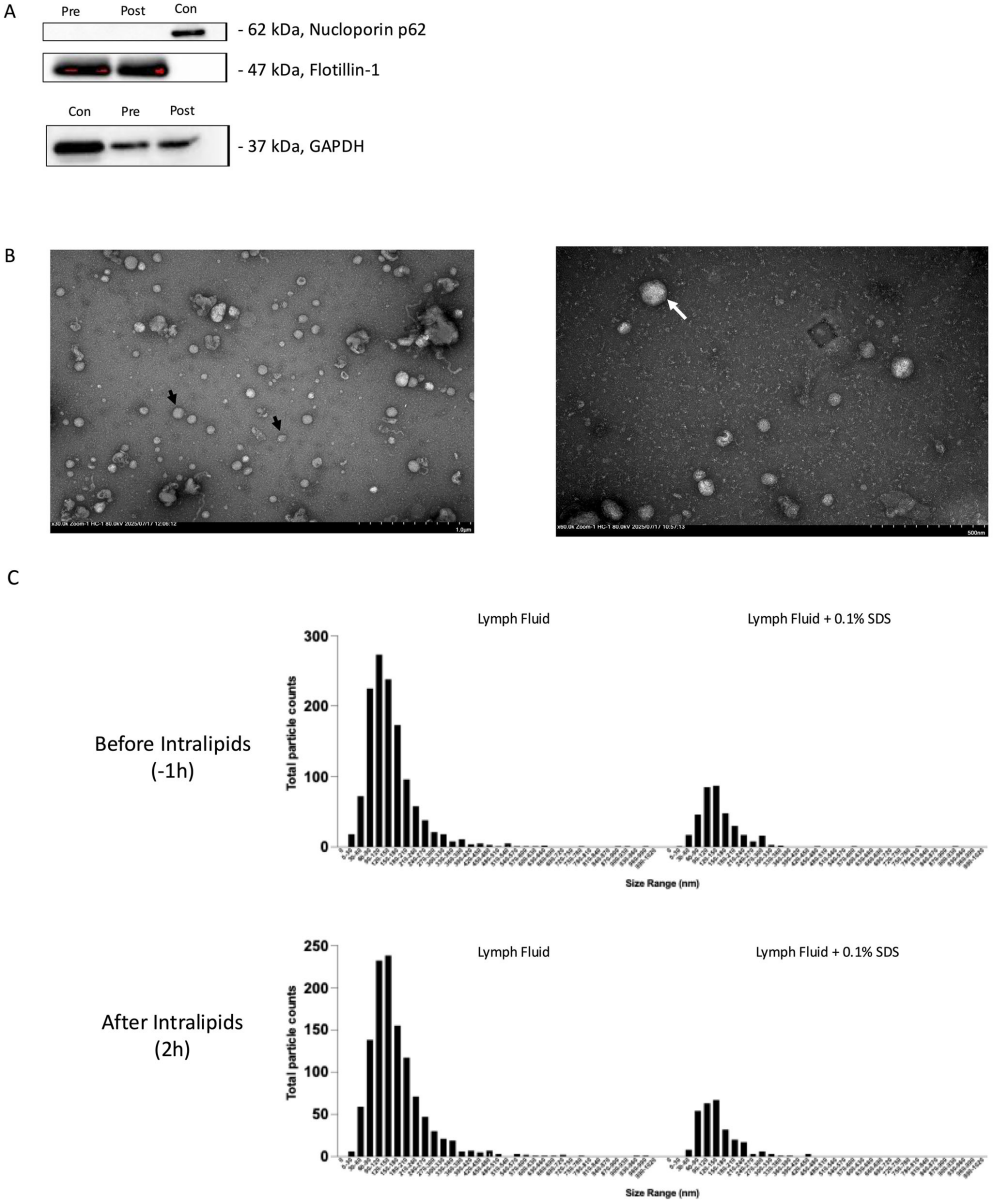
**The intestine secretes EVs into mesenteric lymph**. (A) Western blot of flotillin‐1, nucleoporin p62, and Gapdh. Pre: before Intralipid infusion (–1 h), Post: after Intralipid infusion (2 h), Con: Fibroblast cell control. (B) Transmission electron microscopy of EVs isolated from cell‐free lymph collected before saline infusion, with 30K (left) and 60K (right) magnification. (C) Nanoparticle tracking analysis of EVs isolated from lymph fluid before (top) and after (bottom) lipid infusion, without (left) and with (right) incubation with 0.1% SDS.

To determine whether the intestine secretes EVs, we performed EV isolation using ultracentrifugation from the lymph fluids collected both at the fasted state and after lipid/saline infusion. Western blotting indicated that the EV isolates were positive for the vesicle membrane marker Flotillin‐1. They were positive for the cytosolic protein marker GAPDH, indicating intact vesicles. They did not contain the nuclear marker nucleoporin p62, suggesting free of contamination by intact cells (Figure 2A). Under TEM, vesicle‐like structures were visualized, with sizes in the range of 20 to 300 nm (Figure 2B). This was further confirmed by NTA (Figure 2C), where the majority of particle detected in lymph before and after lipid infusion were found in the size range of 30–300 nm. Moreover, incubating with 0.1% SDS dramatically reduced particle numbers in lymph (from 1.7 × 10^10^ to 4.7 × 10^8^ particles/mL pre‐lipid, from 1.4 × 10^10^ to 3.1 × 10^8^ particles/mL post‐lipid), indicating that the particles detected by NTA were membrane bound vesicles. Since our isolation approach yielded a heterogeneous population containing vesicles across a broad size spectrum, we refrained from applying size‐dependent descriptors such as “small EVs” or “large EVs.”

### 3.3 Lipid Absorption Increases the Secretion of CD63^+^ and CD81^+^ EVs From the Intestine

To further investigate whether EV secretion from the intestine is altered during lipid absorption, we compared the positivity of EV‐associated markers in lymph collected before and after lipid infusion. EV surface markers, such as tetraspanin family proteins CD63, CD81 and CD9, were used to detect the presence of EVs and further define EV subtypes via bead‐based FCM with fluorescently conjugated antibodies against these EV markers. Aldehyde/sulfate latex beads used in our study can couple EVs which facilitate EV analysis by increasing their size and providing a stable scaffold for antibody staining.

In lymph before and after saline or Intralipid infusion, we detected positive CD63, CD81 and CD9 signals when gating at beads population, indicating the presence of EVs in the mesenteric lymph (Figure 3A–C). Similar to lipid output, saline infusion did not significantly affect the levels of CD63 and CD81 positivity in lymph compared with baseline (before saline infusion). The percentage of CD63 and CD81 positivity and their MFI remained relatively unchanged with saline infusion. In contrast, a significant decrease in the percentage of CD9 positivity was observed after saline infusion (*P* < 0.05, Figure 3B,D), although the MFI remained unchanged. Interestingly, lipid infusion significantly increased both the percentages and MFI of CD63 and CD81 positivity (*P* < 0.01, Figure 3C,D). In contrast, although the MFI of CD9 was increased with lipid infusion (*P* < 0.01, Figure 3C,D), the percentage was not significantly affected.

FIGURE 3
**CD63, CD81 and CD9 expression in cell‐free lymph**. (A) Representative pseudocolor plots of cell‐free lymph collected before (–1 h) and 2 h after (2 h) saline or Intralipid infusion. (B) Representative histograms of CD63, CD81 and CD9 expression in negative control, cell‐free lymph collected 1 h before (–1 h) and 2 h (2 h) after saline infusion. (C) Representative histograms of CD63, CD81 and CD9 expression in cell‐free lymph collected 1 h before (–1 h) and 2 h after (2 h) Intralipid infusion. (D) Frequency of parent (%) (a‐c) and Median Fluorescence Intensity (d‐f) for CD63, CD81 and CD9 in cell‐free lymph collected before (–1 h) and 2 h after (2 h) saline or Intralipid infusion. Two‐way ANOVA or mixed‐effects analysis with multiple comparisons followed by Tukey's multiple comparisons test was used for statistical analysis. Results are expressed as mean ± SEM: Saline, *n* = 6 rats; Intralipids, *n* = 6 rats. * *P* < 0.05 and ** *P* < 0.01, Saline vs Intralipid; # *P* < 0.05, ## *P* < 0.01, after Intralipid vs before Intralipid.

**Figure:**
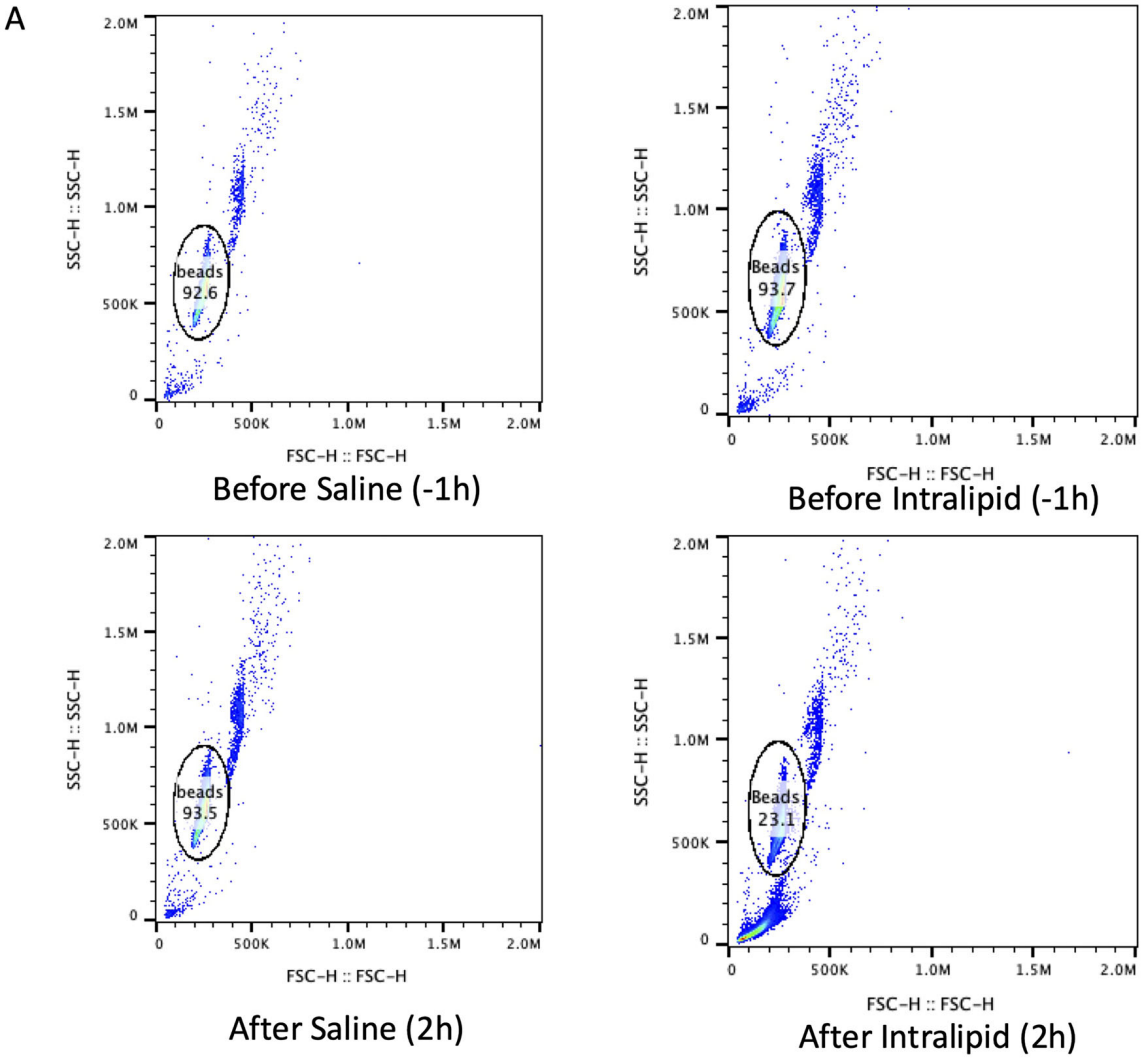

**Figure:**
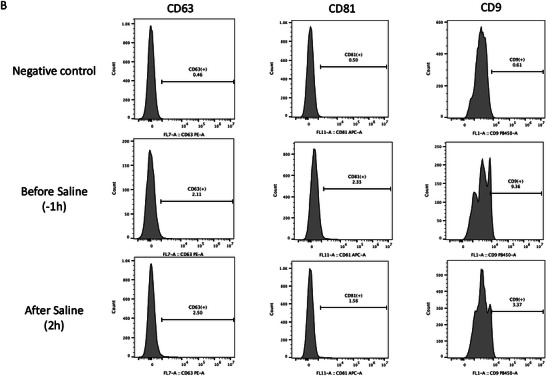

**Figure:**
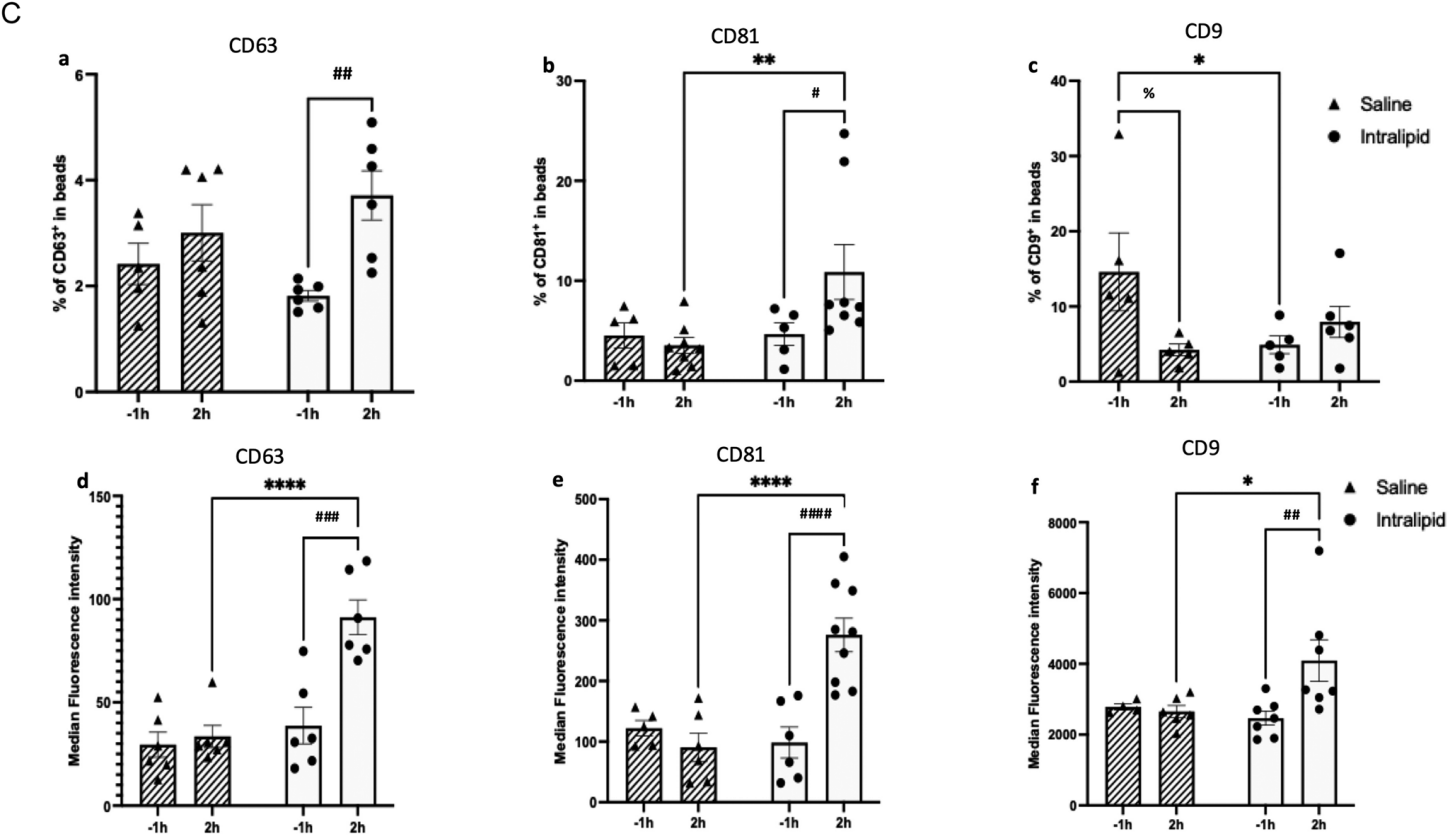

**Figure:**
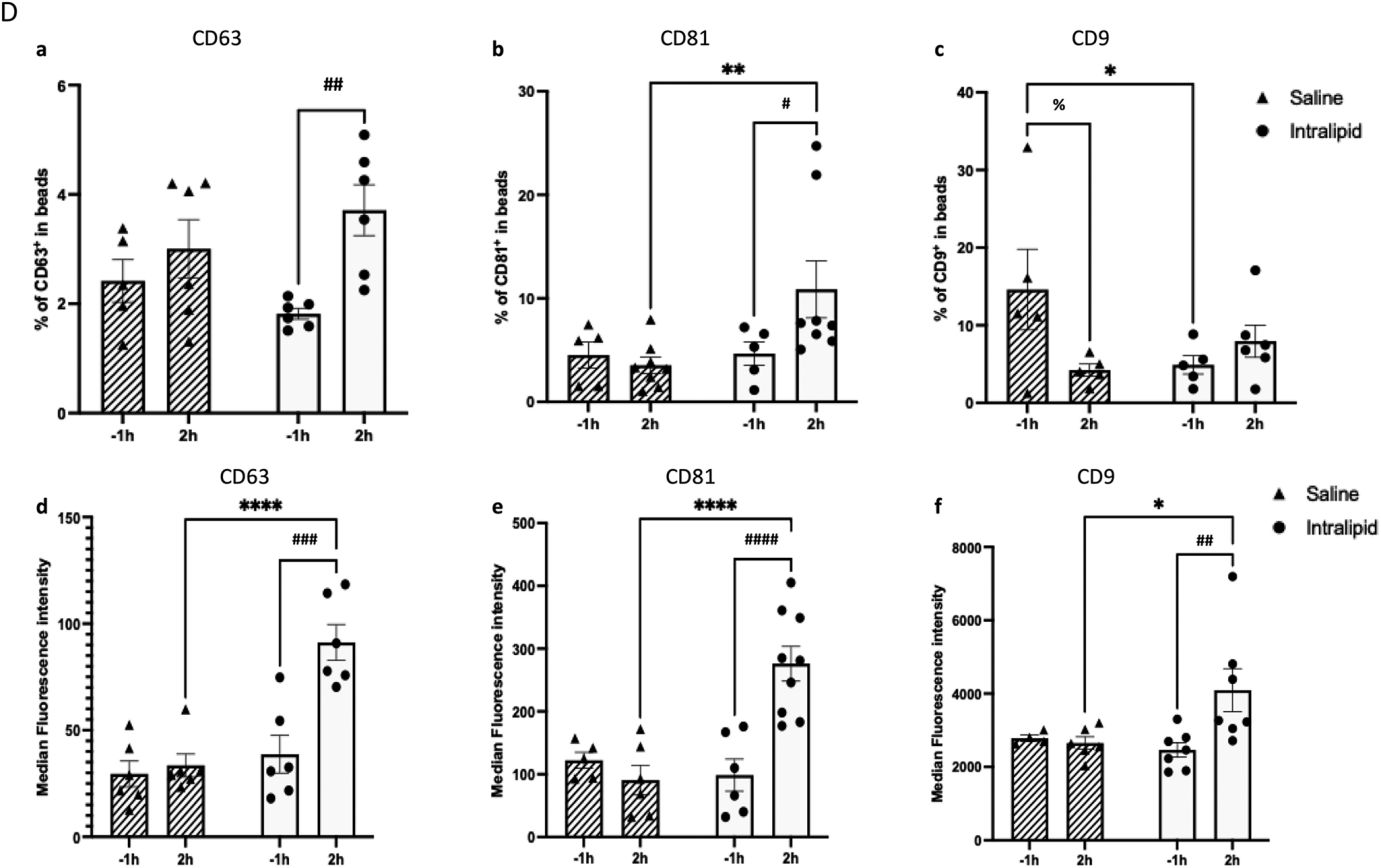

### 3.4 Chylomicron Depletion Has Differing Effects on CD63^+^, CD81^+^ and CD9^+^ EVs

**FIGURE 4:**
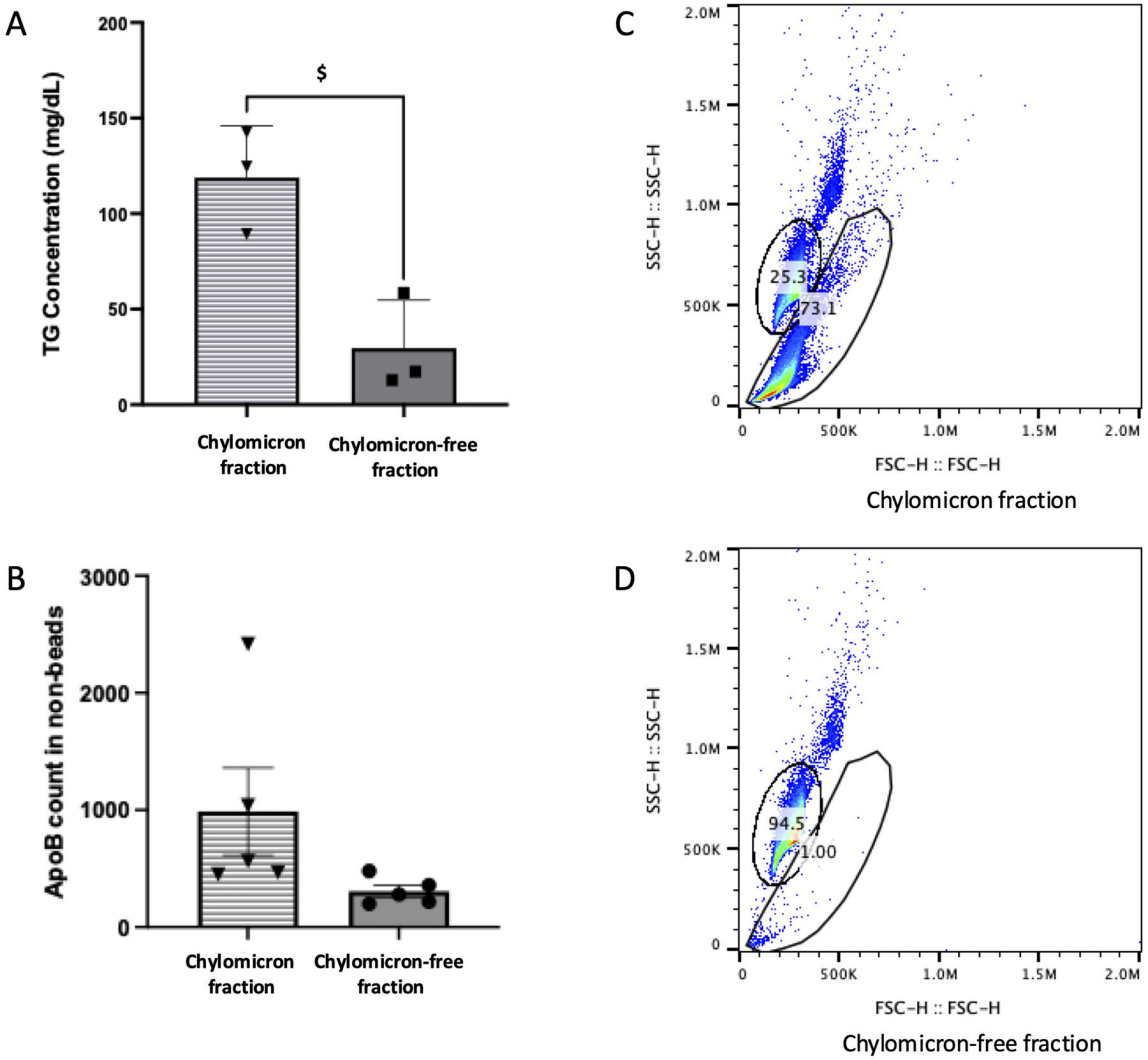
**Chylomicron depletion on EV purity**. (A) TG concentrations in chylomicron fractions and chylomicron‐free fractions following chylomicron depletion by ultracentrifugation. *n* = 3 rats. $ *P* < 0.05. (B) ApoB count in chylomicron fractions and chylomicron‐free fractions in non‐beads population following chylomicron depletion by ultracentrifugation. *n* = 5 rats. (C) Representative pseudocolor plots of chylomicron fractions and chylomicron‐free fractions following chylomicron depletion of cell‐free lymph collected 2 h after Intralipid infusion with the frequency of parent (%) for different populations.

**FIGURE 5:**
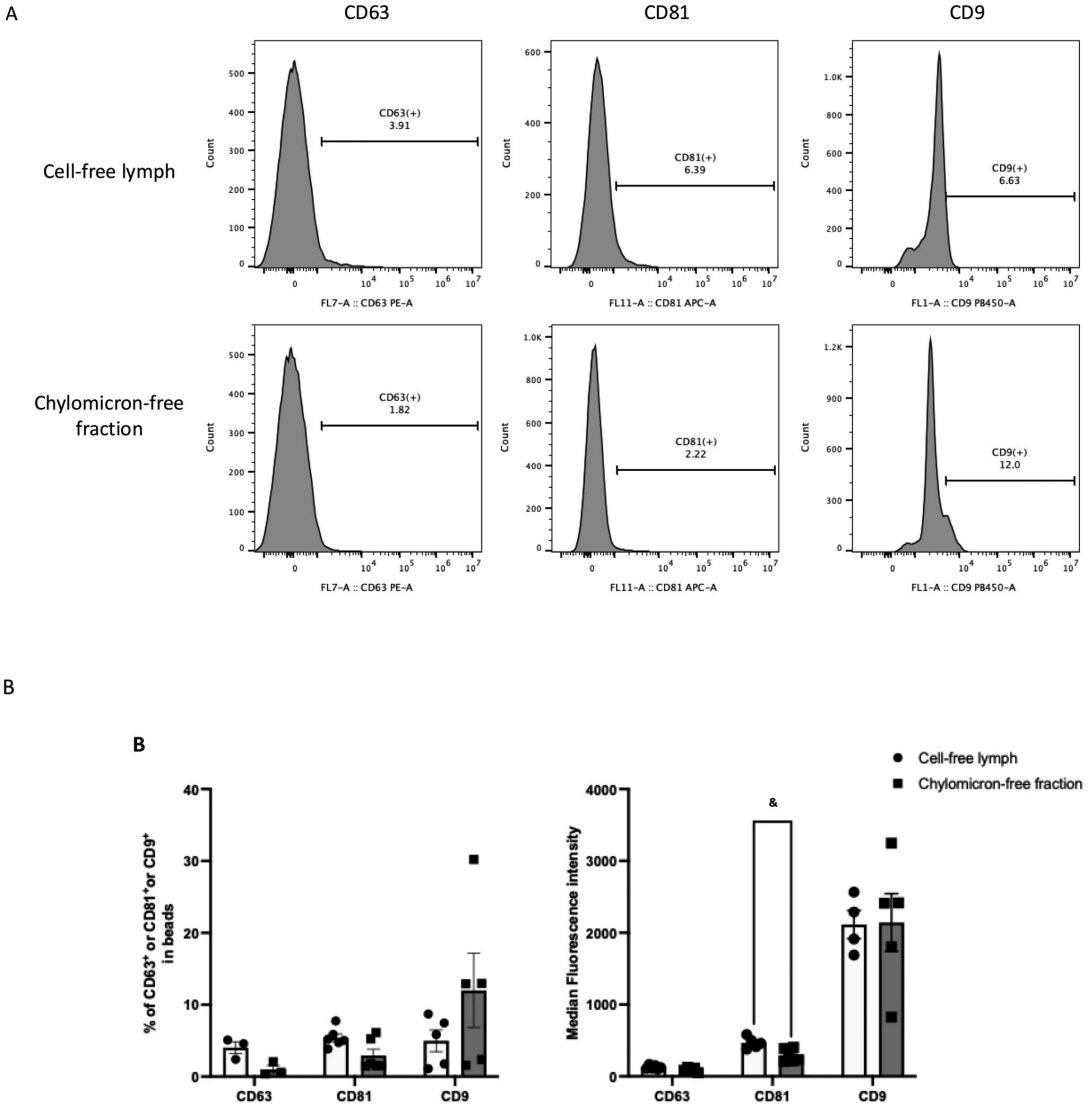
**CD63, CD81 and CD9 expression before and after chylomicron depletion**. (A) Representative histograms of CD63, CD81 and CD9 expression in cell‐free lymph collected 2 h after Intralipid infusion (2 h) and in chylomicron‐free fractions after chylomicron depletion. (B) Frequency of parent (%) (left) and Median Fluorescence Intensity (right) for CD63, CD81 and CD9 in cell‐free lymph collected 2 h after Intralipid infusion (2 h) and in chylomicron‐free fractions after chylomicron depletion. Two‐way ANOVA or mixed‐effects analysis with multiple comparisons followed by Tukey's multiple comparisons test was used for statistical analysis. Results are expressed as mean ± SEM. *n* = 5 rats. & *P* < 0.05 cell‐free lymph vs chylomicron‐free fraction.

Interestingly, we observed a unique and large unknown particle population in the samples after lipid infusion compared with the samples collected either during the fasted state (prior to infusion) or following saline infusion, and this unknown population appears to be the predominant population (Figure 3A). Since lipid infusion increases chylomicron secretion into the lymph, we hypothesized that the appearance of this population following lipid infusion was due to EV association with chylomicron particles. To test this hypothesis, we separated the cell‐free lymph samples collected after a lipid load into chylomicron and chylomicron‐free fractions. As expected, chylomicron fraction was enriched with TG and ApoB compared with chylomicron‐free fraction (Figure 4A,B). It is noted that ApoB was still detectable, indicating incomplete removal of chylomicrons. Indeed, chylomicron depletion dramatically decreased the unknown particle population observed in the after‐lipid lymph samples (Figure 4C,D). These results indicate that chylomicrons made up the majority of this unknown population in the lipid‐rich samples. Chylomicron depletion tended to increase the percentages of CD9 positivity and decrease CD63 and CD81 positivity, with significant increase in CD81 MFI, when compared to those in the cell‐free lymph (Figure 5A,B). These results indicated that chylomicron depletion had differing effects on CD63^+^, CD81^+^ and CD9^+^ EVs.

### 3.5 CD63, CD81 And CD9 Expressions Differ on ApoB+ Particles

Interestingly, the chylomicron fraction remained to contain CD63^+^, CD81^+^ and CD9^+^ particles (Figure 6A). These particles theoretically could include both lighter EVs and EVs associated with chylomicrons. To identify EV‐chylomicron association, we examined the expression of EV markers CD63, CD81 and CD9 on the particles positive with chylomicron marker ApoB. CD63 and CD81, but not CD9, were detected on ApoB^+^ particles in both cell‐free lymph and chylomicron fractions (Figure 6B,C). In addition, although not statistically significant, chylomicron depletion tended to increase the percentage of CD81 positivity and the MFI of CD63 on ApoB+ particles (Figure 6C). These results suggest differential associations of EV sub‐types with chylomicrons, and it is possible that the removal of chylomicrons also removed certain types of EVs.

FIGURE 6
**CD63, CD81, CD9 expression on ApoB positive particles in chylomicron fraction**. (A) Representative histograms of CD63, CD81 and CD9 expression on ApoB positive particles in negative control and in chylomicron fractions. (B) Representative histograms of CD63, CD81 and CD9 expression within ApoB positive particles in negative control, in cell‐free lymph collected 2 h after Intralipid infusion, and in chylomicron fractions. (C) Frequency of parent (%) (left) and Median Fluorescence Intensity (right) for CD63, CD81 and CD9 within ApoB positive population in negative control, in cell‐free lymph collected 2 h after intralipid infusion, and in chylomicron fractions. Two‐way ANOVA or mixed‐effects analysis with multiple comparisons followed by Tukey's multiple comparisons test was used for statistical analysis. Results are expressed as mean ± SEM. *n* = 6 rats. @ *P* < 0.05, @@ *P* < 0.01 CD63 vs CD9; ^ *P* < 0.05 CD81 vs CD9.

**Figure:**
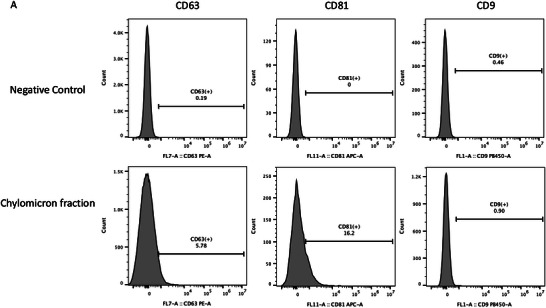

**Figure:**
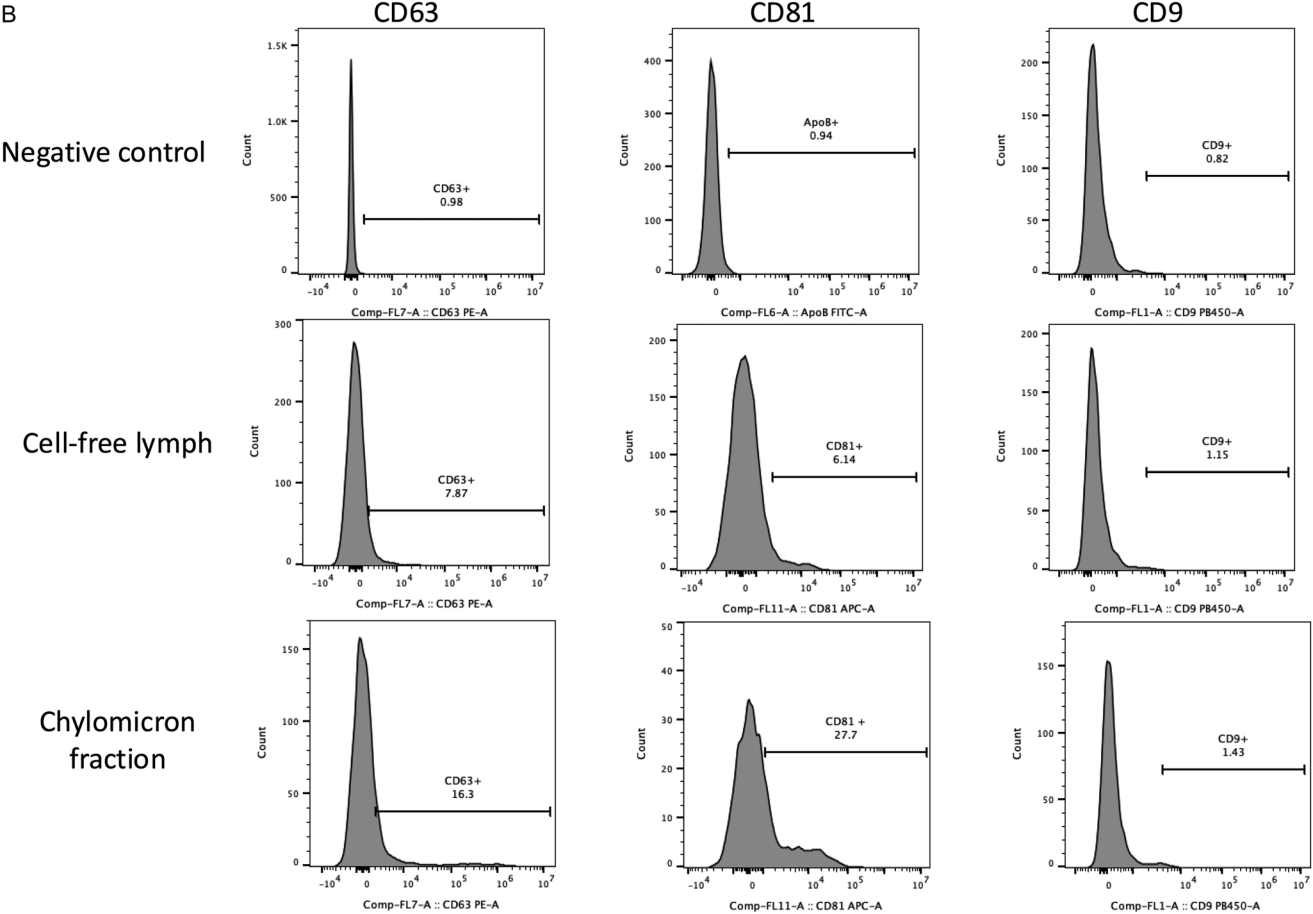

**Figure:**
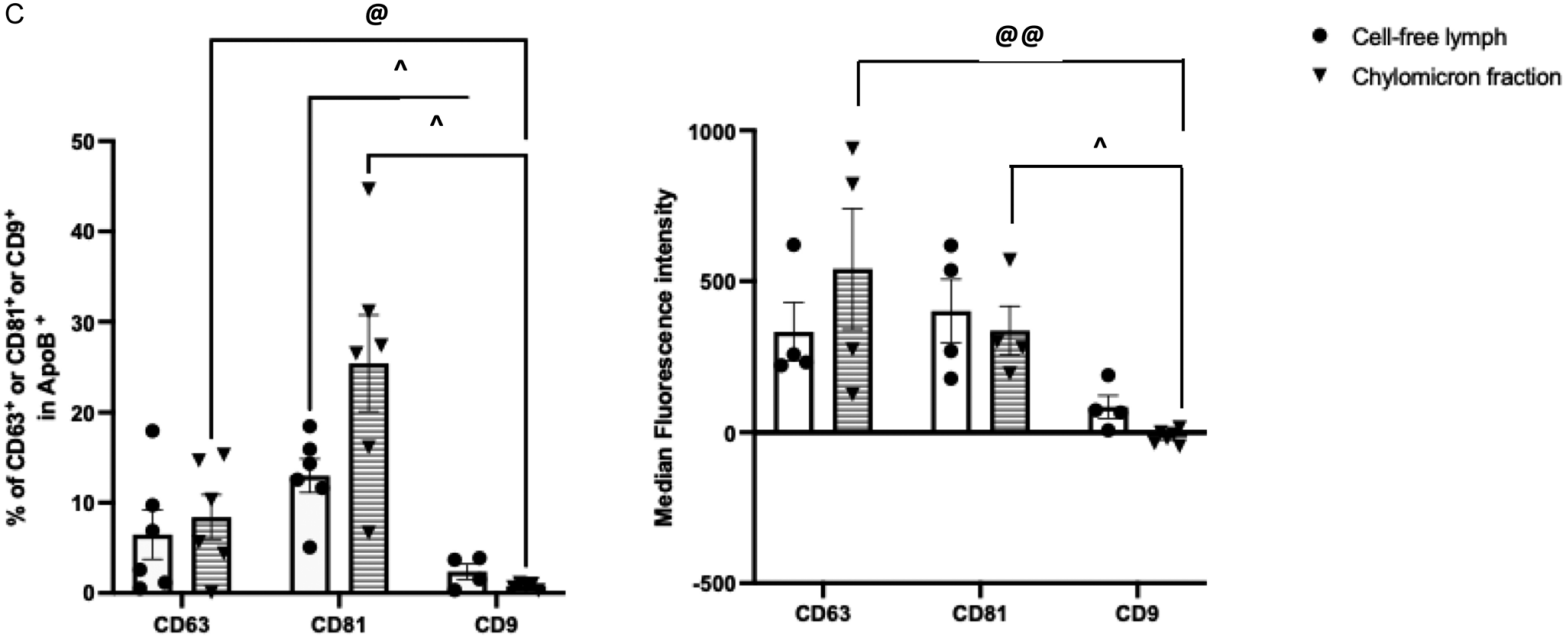

## 4 Discussions

EVs mediate cell‐to‐cell communication and, owing to their potential as disease markers and effectors, their secretions by various organs have been investigated. The intestine, vital for normal physiology and systemic health with its diverse functions and secretions, has not been investigated for its EV secretion on the organ level. Here we examined the presence of EVs in the mesenteric lymph, a biofluid containing intestinal secretions. We took advantage the mesenteric lymph duct cannulated rat model that enables direct collection of intestinal secretions and offers a liquid biopsy of the entire intestine. We examined EV‐associated markers in lymph at fasting and during lipid absorption, an important physiological process that utilizes the mesenteric lymph duct as the secretory path. We demonstrate that EVs are secreted from the intestine into the mesenteric lymph, that EV secretion is altered during lipid absorption, and that EV subtypes may exhibit differential interactions with chylomicrons.

EV secretions have been examined on organ/tissue level for the liver, adipose, and muscle, and in biofluids such as blood, urine, and milk (Miotto et al. 2024, Liu et al. 2025, Haque et al. 2025); however, intestine EV secretion on the organ level has not been investigated. The mesenteric lymph duct and portal vein are the two primary pathways for absorbing and transporting nutrients, metabolites, and fluids from the gastrointestinal tract to the systemic circulation (Yu et al. 2022). Complex dietary fats, especially long‐chain fatty acids, are packaged into lipoprotein particles (chylomicrons) and secreted into mesenteric lymph. Mesenteric lymph also collects certain gut hormone secretions. For instance, glucagon‐like peptide‐1 levels are several folds higher in mesenteric lymph than in circulating blood due to low levels of dipeptidyl peptidase 4 (the cleavage enzyme) in mesenteric lymph (D'Alessio et al. 2007, Ohlsson et al. 2014, Jejelava et al. 2018). Previously, an in vitro study showed the release of exosome‐like vesicles with 30‐90‐nm diameters from the apical and basolateral sides of human intestinal epithelial cells (van Niel et al. 2001). We speculate that mesenteric lymph may contain specific types of EVs and may serve as a reservoir of EVs protected from certain modifications.

Mesenteric lymph is a biofluid rich with lipoproteins during fat ingestion. FCM using antibodies against CD63, CD9 and CD81, known EV markers for detection and immunocapture, allowed identification of EVs in the presence of lipoproteins. Using FCM, we were able to detect CD63, CD81 and CD9, tetraspanins enriched in EVs, in lymph, indicating EV presence in mesenteric lymph. Since the mesenteric lymph duct mainly drains the intestine, these EVs reflects the secretion from the intestine, although minor amount of EVs originated from other parts of the body may also enter. In addition, we found that lipid absorption or lipoprotein secretion increased the signals of EV‐associated markers in lymph. The exact mechanisms are not clear; it may be related to the rapid adaption of the intestine to nutrient ingestion. The intestine responds to short‐term dietary interventions, such as high‐fat feeding, with cellular and molecular adaptions (Enriquez et al. 2022). A cyclic reciprocal relationship has been shown for IL‐22 secretion and lipid absorption in mice during the feeding and fasting cycle (Talbot et al. 2020). We observed several differences among EV‐associated markers in their response to lipid/saline infusion. For instance, the positive signal of CD9 decreased following saline infusion and tended to increase following lipid infusion, while the positive signal of CD63 tended to increase after saline and lipid infusion. Interestingly, even the positive signal of CD9 decreased, it remained higher than that of CD63. These results indicating that subtype of EVs may have different responses to physiological processes. This may be due to different functions of tetraspanin proteins in EV biogenesis. Recently, Duke et al. showed CD9 affects the regulation and secretion of CD63 (Duke et al. 2025). Alternatively, these differences may relate to the state of cells. Kormelink et al. demonstrated marked differences in CD9 and CD63 contents of EV derived from unstimulated or degranulated mast cells (Groot Kormelink et al. 2016). Future studies are required to investigate the mechanisms of intestinal EV secretion during lipid absorption. In addition, since the intestine absorbs glucose, peptides and shorter chain fatty acids directly into the portal vein, future studies are needed to evaluate EV secretions from the intestine into both lymph and portal blood and in response to other nutrients.

We are fully aware of the challenges associated with isolating EVs, particularly from lipid‐rich samples. Lipoproteins are defined as non‐vesicle extracellular particles (Welsh et al. 2024). Since lipoproteins and EVs share many biochemical properties, including size range, density, and molecular composition, the separation of lipoproteins and EVs is inheritably associated with technical difficulties (Welsh et al. 2024). Furthermore, it has been suggested that certain molecules (such as proteins and lipids) that co‐isolate with EVs may be crucial for the biological functions of EVs and that these molecules should not be considered as contamination. For instance, Tóth et al. reported protein corona structures with plasma derived EVs (Tóth et al. 2021). Similarly, Radeghieri et al. reported the formation of “EV‐protein corona” on plasma EVs that differed between healthy and antithrombin qualitative deficiency‐affected subjects (Radeghieri et al. 2022). Recently, Brachyahu et al. reported a reduction in circulating CD63^+^ EVs in mice with increased low‐density lipoprotein levels, suggesting a shift of specific EVs towards the formation of EV‐lipoprotein complex (Kestecher et al. 2024). In our current study, we performed co‐localization of chylomicron marker (ApoB) and EV markers in the chylomicron fraction. In the ApoB‐positive population, the presence of CD63 and CD81 suggests possible association of EVs with chylomicrons. Moreover, undetectable CD9 on the ApoB‐positive population suggests EVs may have different affinities to chylomicrons. Indeed, MISEV recognizes the limitations of using tetraspanin‐based immunocapture for EV enrichment (Welsh et al. 2024). Proteomics analysis of heterogeneous EV populations in cell culture medium identified EVs co‐enriched with CD63, CD9 and CD81 and EVs devoid of CD63 and CD81 but enriched in CD9 (Kowal et al. 2016), which further supports our findings on EV sub‐types. In our study, following an extra step to remove chylomicrons, we observed differential changes among EV markers, which suggests different affinities of EV subtypes to chylomicrons. Future studies are warranted to further characterize specific EV subtypes and their relationship to chylomicrons.

In summary, this study took advantage of a specialized technique for mesenteric lymph collection to establish that EVs are present in the mesenteric lymph fluid and that EV secretion from the intestine can quickly respond to lipid absorption. Further, gut‐derived EVs secreted during lipid absorption may have multiple subtypes with different affinities to chylomicrons under normal physiological conditions. These findings open a venue for future studies to examine gut secretion of EVs in other conditions, such as certain pathologies, and to characterize the biological functions of gut‐derived EVs.

## Author Contributions

**Tianyu Hang**: conceptualization, investigation, project administration, formal analysis, visualization, writing – original draft. **Rita Wang**: investigation. **Kundanika Mukherjee**: investigation. **Uday Sandhu**: investigation. **Fengxia Xiao**: investigation, visualization, formal analysis. **Dylan Burger**: resources, supervision, writing – review and editing. **Changting Xiao**: conceptualization, resources, funding acquisition, supervision, writing – review and editing.

## Conflicts of Interest

The authors declare no conflict of interest.

## Supporting information




**Supporting Figure 1: Transmission electron microscopy images of EV isolated from lymph after lipid infusion**. (A) TEM of chylomicron fractions following chylomicron depletion of cell‐free lymph collected 2 h after lipid infusion with 30K magnification. (B) TEM of EVs isolated from chylomicron‐free fractions following chylomicron depletion of cell‐free lymph collected 2 h after lipid infusion with 30K magnification.

**Supporting Figure 2A: CD63, CD81 and CD9 expression in cell‐free lymph**. (A) Representative pseudocolor plots and histograms of CD63, CD81 and CD9 in chylomicron‐free fractions following chylomicron depletion of lymph collected 2 h after lipid infusion with the frequency of parent (%) for beads populations (left), and after incubating with 0.05% Triton‐X (right). (B) Frequency of parent (%) for CD63, CD81 and CD9 in chylomicron‐free fractions after chylomicron depletion in cell‐free lymph collected 2 h after lipid infusion with and without 0.05% Triton‐X incubation. Welch's T test was used for statistical analysis. Results are expressed as mean ± SEM. *n* = 5 rats. * *P* < 0.05 lymph vs lymph incubated with 0.05% Triton‐X.

Supporting Figure 2B

**Supporting Figure 3: Representative plots of gating strategies**. (A) Gating strategies used in FlowJo. (B) Representative plots for gating from samples: beads (left), beads only control (middle, right) are shown.

**Supporting Figure 4A: CD63, CD81 and CD9 expression in cell‐free lymph**. (A) Representative pseudocolor plots of CD63, CD81 and CD9 expression in negative control, cell‐free lymph collected 1 h before saline infusion (−1 h) and 2 h after saline infusion (2 h). (B) Representative pseudocolor plots of CD63, CD81 and CD9 expression in cell‐free lymph collected 1 h before (−1 h) and 2 h after (2 h) Intralipid infusion.

Supporting Figure 4B

**Supporting Figure 5: CD63, CD81 and CD9 expression before and after chylomicron depletion**. (A) Representative pseudocolor plots of CD63, CD81 and CD9 expression in cell‐free lymph collected 2 h after Intralipid infusion and in chylomicron‐free fractions after chylomicron depletion.

**Supporting Figure 6: CD63, CD81, CD9 and ApoB expression in chylomicron fraction**. Representative pseudocolor plots of CD63, CD81 and CD9 expression in negative control and in chylomicron fractions.

**Supporting Figure 7A: CD63, CD81, CD9 expression on ApoB^+^ particles in chylomicron fraction**. Representative pseudocolor plots of CD63 (A), CD81 (B) and CD9 (C) expression, and gating stratergies in negative control (unstained control and ApoB stained only sample), cell‐free lymph collected 2 h after lipid infusion, and in chylomicron fractions within ApoB positive population. (D) Representative gating strategies screen shot when using FlowJo for negative control (unstained control and ApoB stained only sample).

Supporting Figure 7B

Supporting Figure 7C

Supporting Figure 7D

## Data Availability

The data that support the findings of this study are available from the corresponding author upon reasonable request.
