# Supplementary figures and images for: Secretion of Extracellular Vesicles Into the Mesenteric Lymph During Fasting and Lipid Absorption
Source: J Extracell Biol. 2026 Jul 23;5(7):e70170. doi: 10.1002/jex2.70170 (PMC13393292; doi:10.1002/jex2.70170)

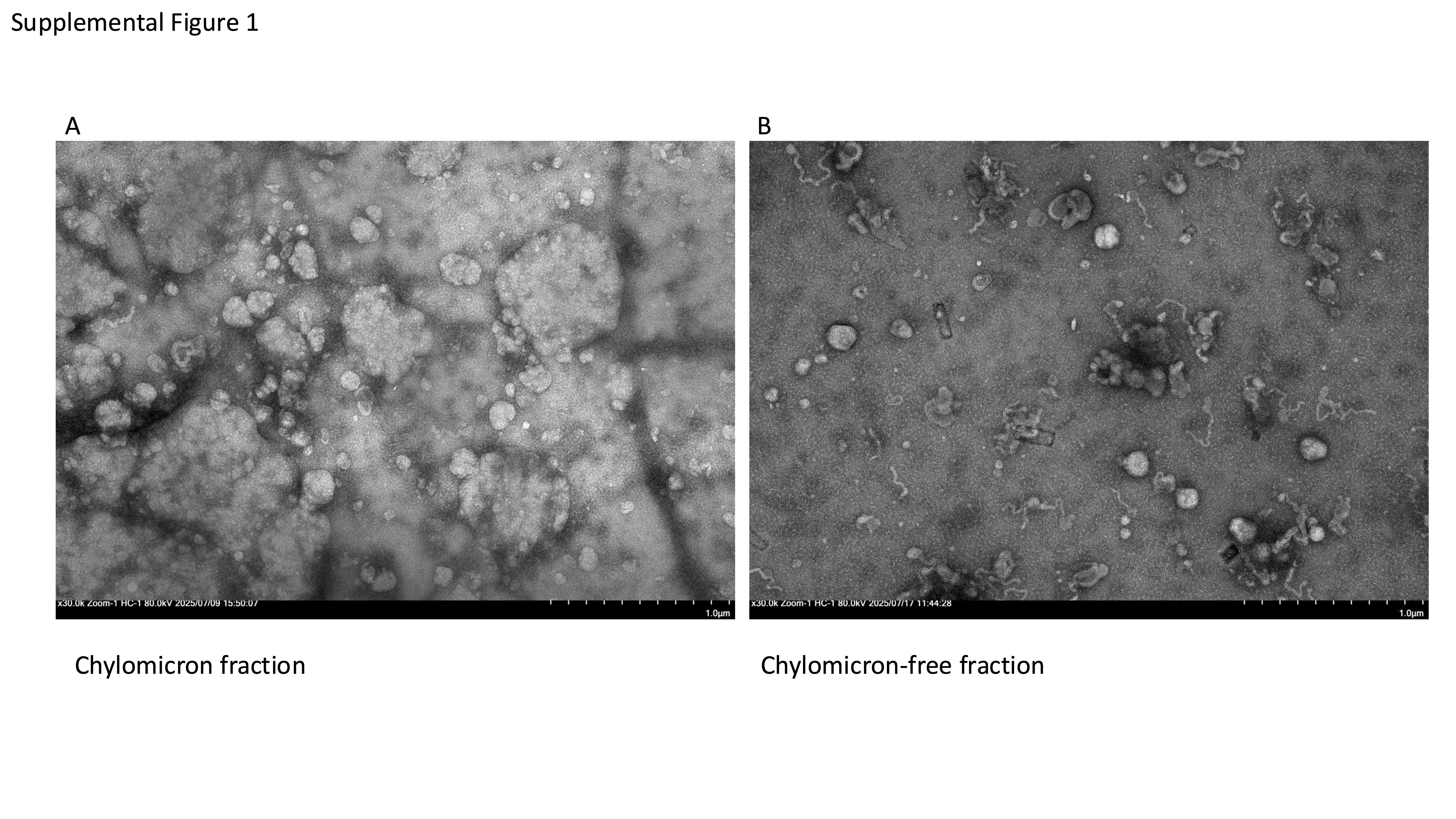

Supplement: Supplementary file 1 — Supporting Figure 1: Transmission electron microscopy images of EV isolated from lymph after lipid infusion. (A) TEM of chylomicron fractions following chylomicron depletion of cell‐free lymph collected 2 h after lipid infusion with 30K magnification. (B) TEM of EVs isolated from chylomicron‐free fractions following chylomicron depletion of cell‐free lymph collected 2 h after lipid infusion with 30K magnification. [file JEX2-5-e70170-s001.jpg]

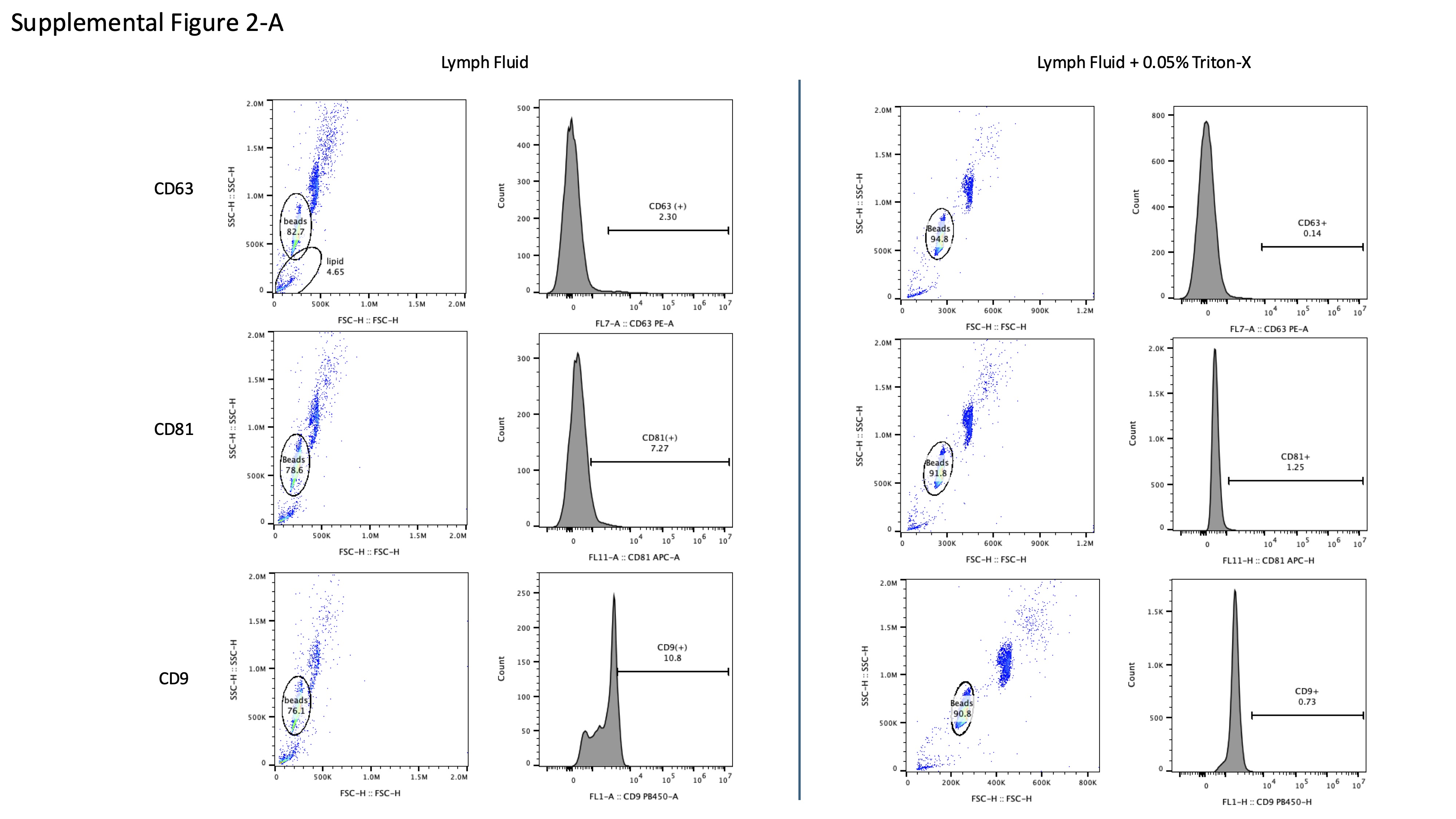

Supplement: Supplementary file 2 — Supporting Figure 2A: CD63, CD81 and CD9 expression in cell‐free lymph. (A) Representative pseudocolor plots and histograms of CD63, CD81 and CD9 in chylomicron‐free fractions following chylomicron depletion of lymph collected 2 h after lipid infusion with the frequency of parent (%) for beads populations (left), and after incubating with 0.05% Triton‐X (right). (B) Frequency of parent (%) for CD63, CD81 and CD9 in chylomicron‐free fractions after chylomicron depletion in cell‐free lymph collected 2 h after lipid infusion with and without 0.05% Triton‐X incubation. Welch's T test was used for statistical analysis. Results are expressed as mean ± SEM. n = 5 rats. * P < 0.05 lymph vs lymph incubated with 0.05% Triton‐X. [file JEX2-5-e70170-s008.jpg]

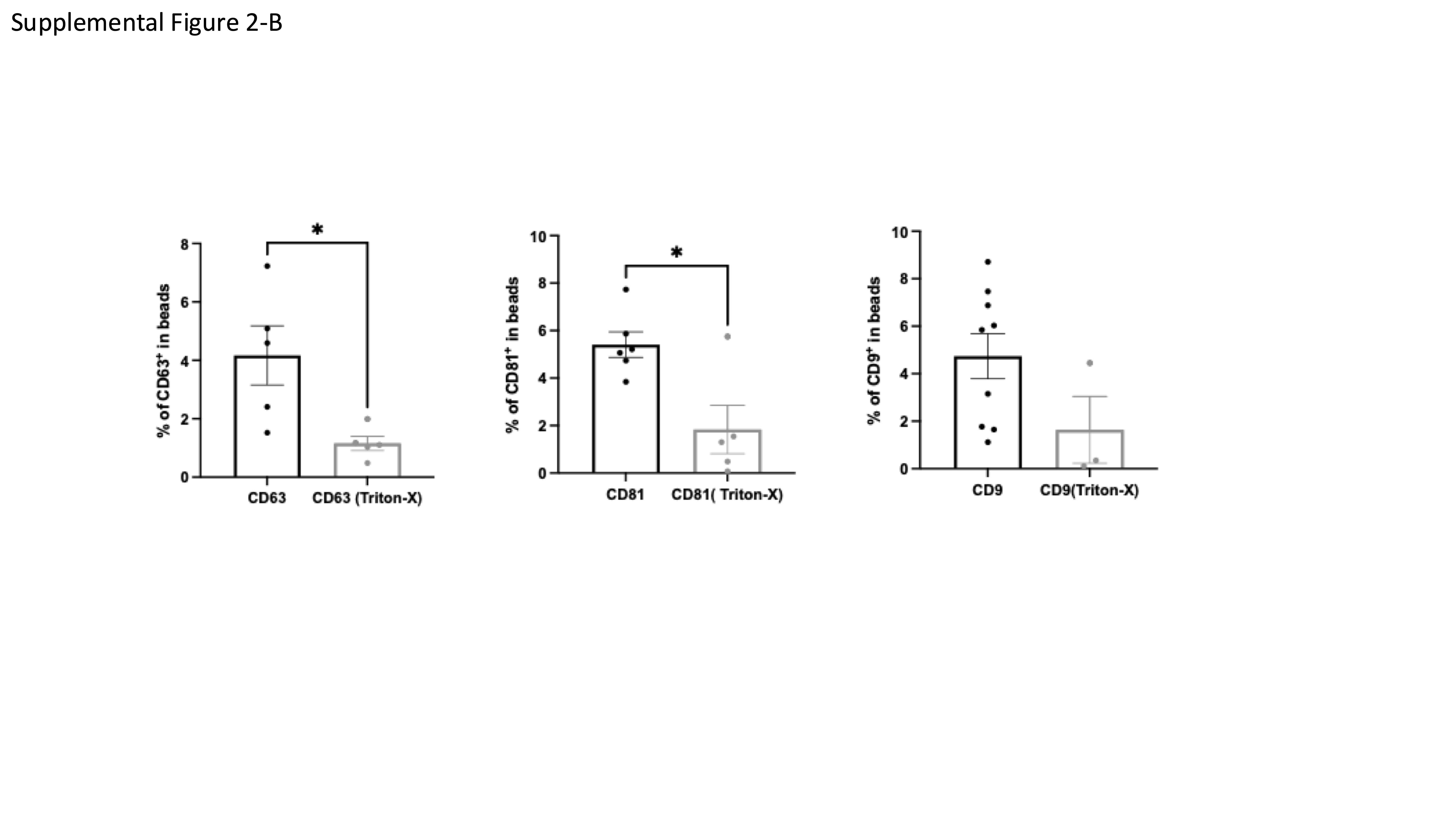

Supplement: Supplementary file 3 — Supporting Figure 2B [file JEX2-5-e70170-s002.jpg]

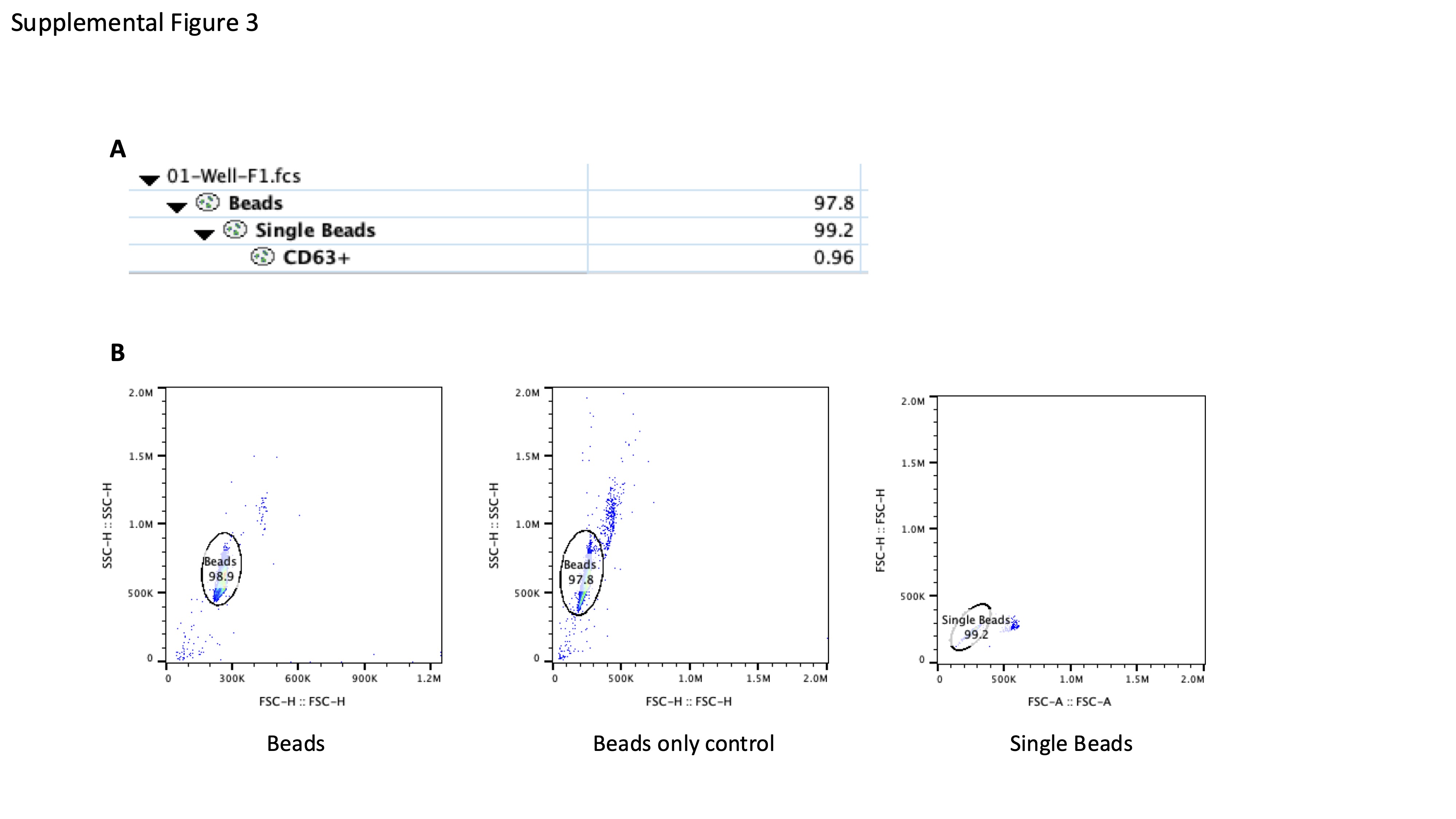

Supplement: Supplementary file 4 — Supporting Figure 3: Representative plots of gating strategies. (A) Gating strategies used in FlowJo. (B) Representative plots for gating from samples: beads (left), beads only control (middle, right) are shown. [file JEX2-5-e70170-s009.jpg]

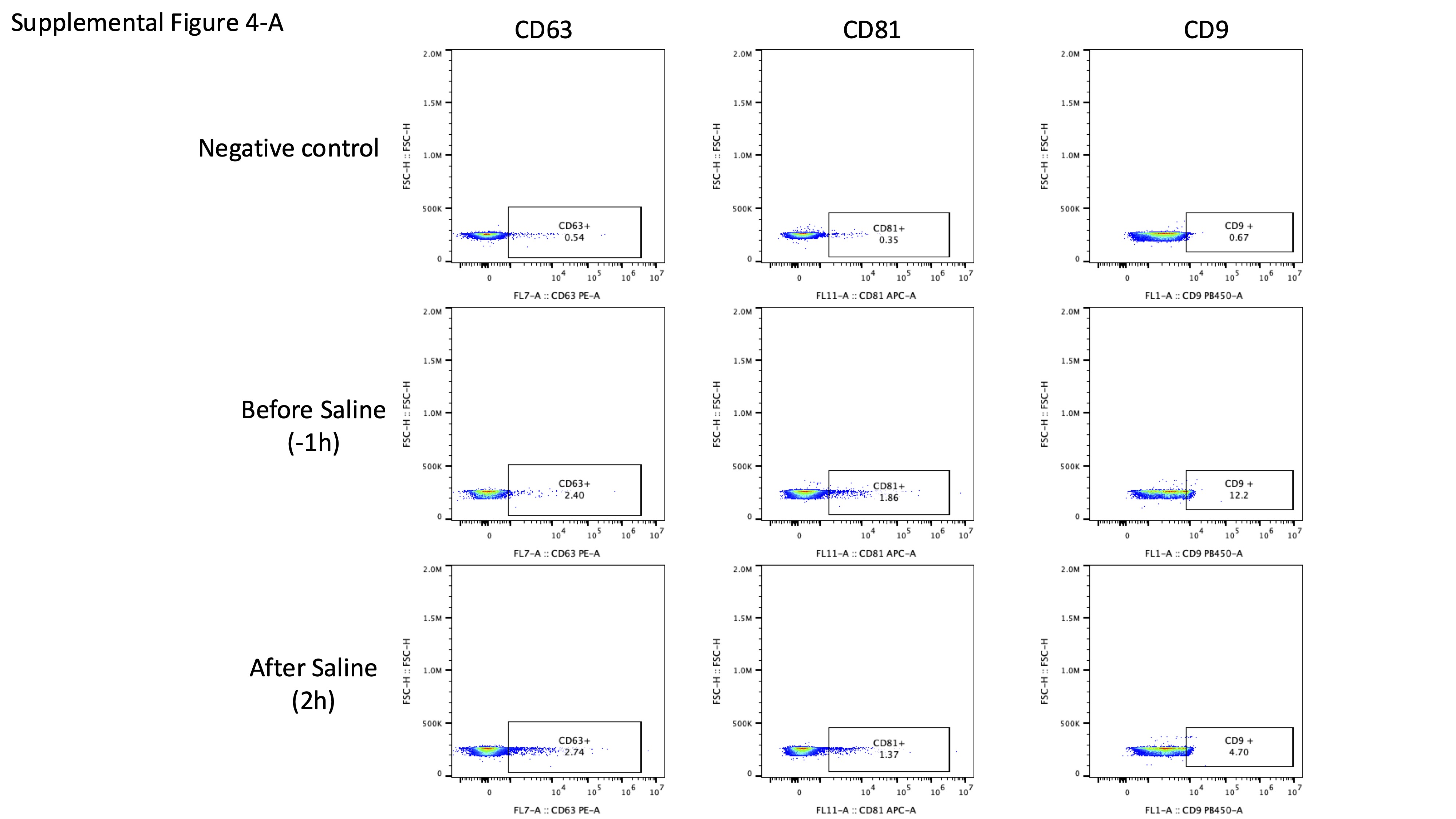

Supplement: Supplementary file 5 — Supporting Figure 4A: CD63, CD81 and CD9 expression in cell‐free lymph. (A) Representative pseudocolor plots of CD63, CD81 and CD9 expression in negative control, cell‐free lymph collected 1 h before saline infusion (−1 h) and 2 h after saline infusion (2 h). (B) Representative pseudocolor plots of CD63, CD81 and CD9 expression in cell‐free lymph collected 1 h before (−1 h) and 2 h after (2 h) Intralipid infusion. [file JEX2-5-e70170-s006.jpg]

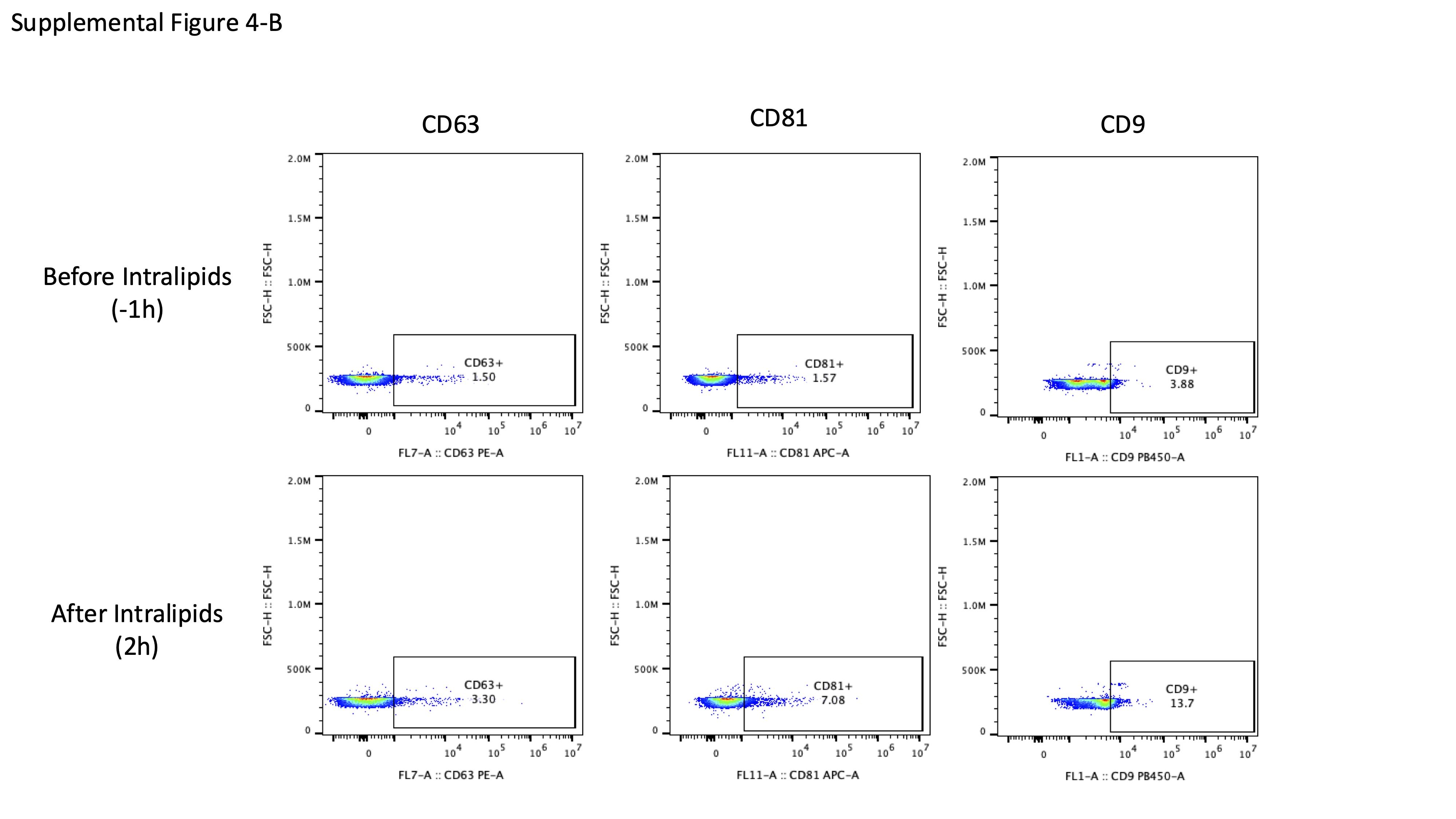

Supplement: Supplementary file 6 — Supporting Figure 4B [file JEX2-5-e70170-s003.jpg]

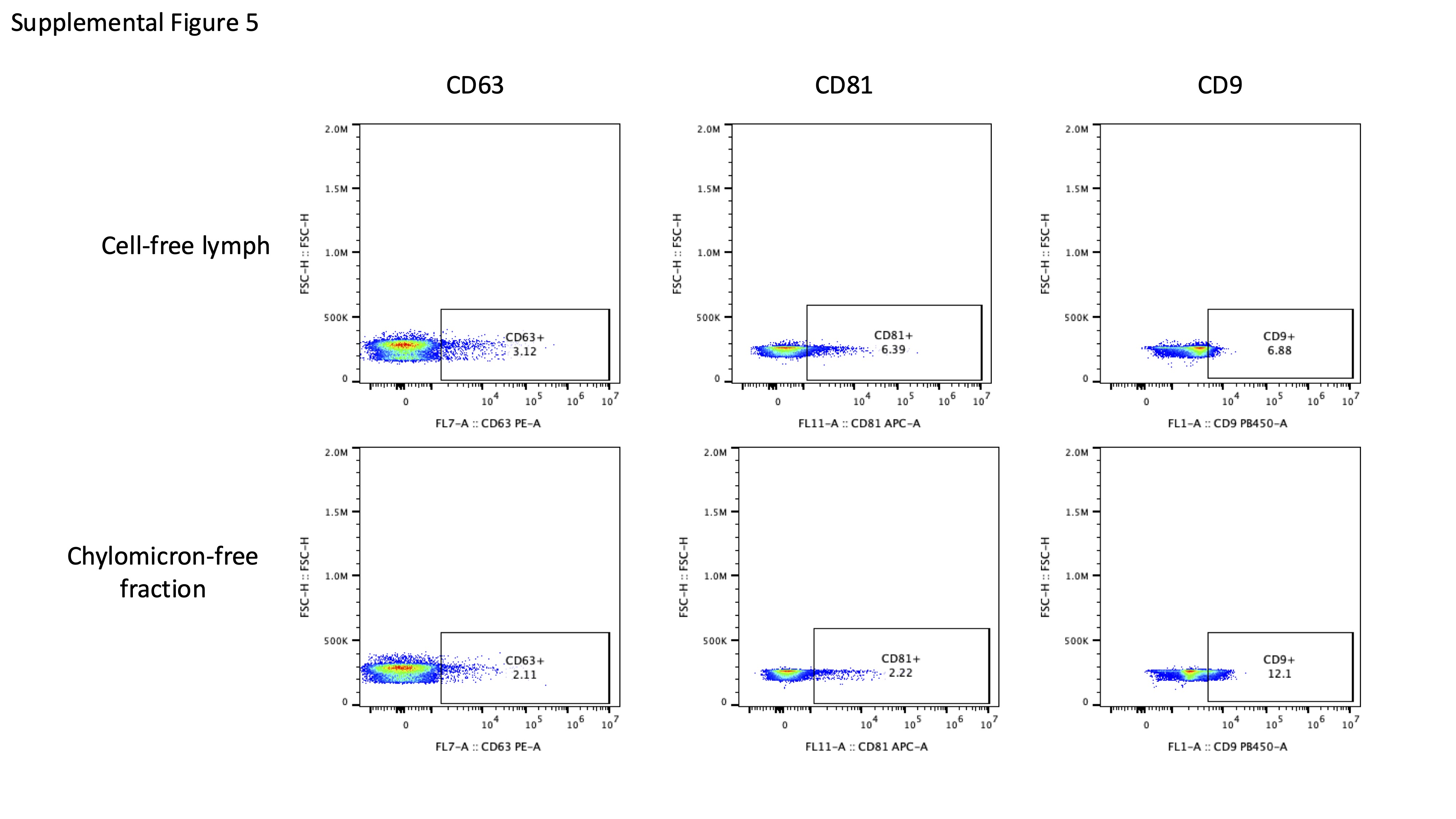

Supplement: Supplementary file 7 — Supporting Figure 5: CD63, CD81 and CD9 expression before and after chylomicron depletion. (A) Representative pseudocolor plots of CD63, CD81 and CD9 expression in cell‐free lymph collected 2 h after Intralipid infusion and in chylomicron‐free fractions after chylomicron depletion. [file JEX2-5-e70170-s010.jpg]

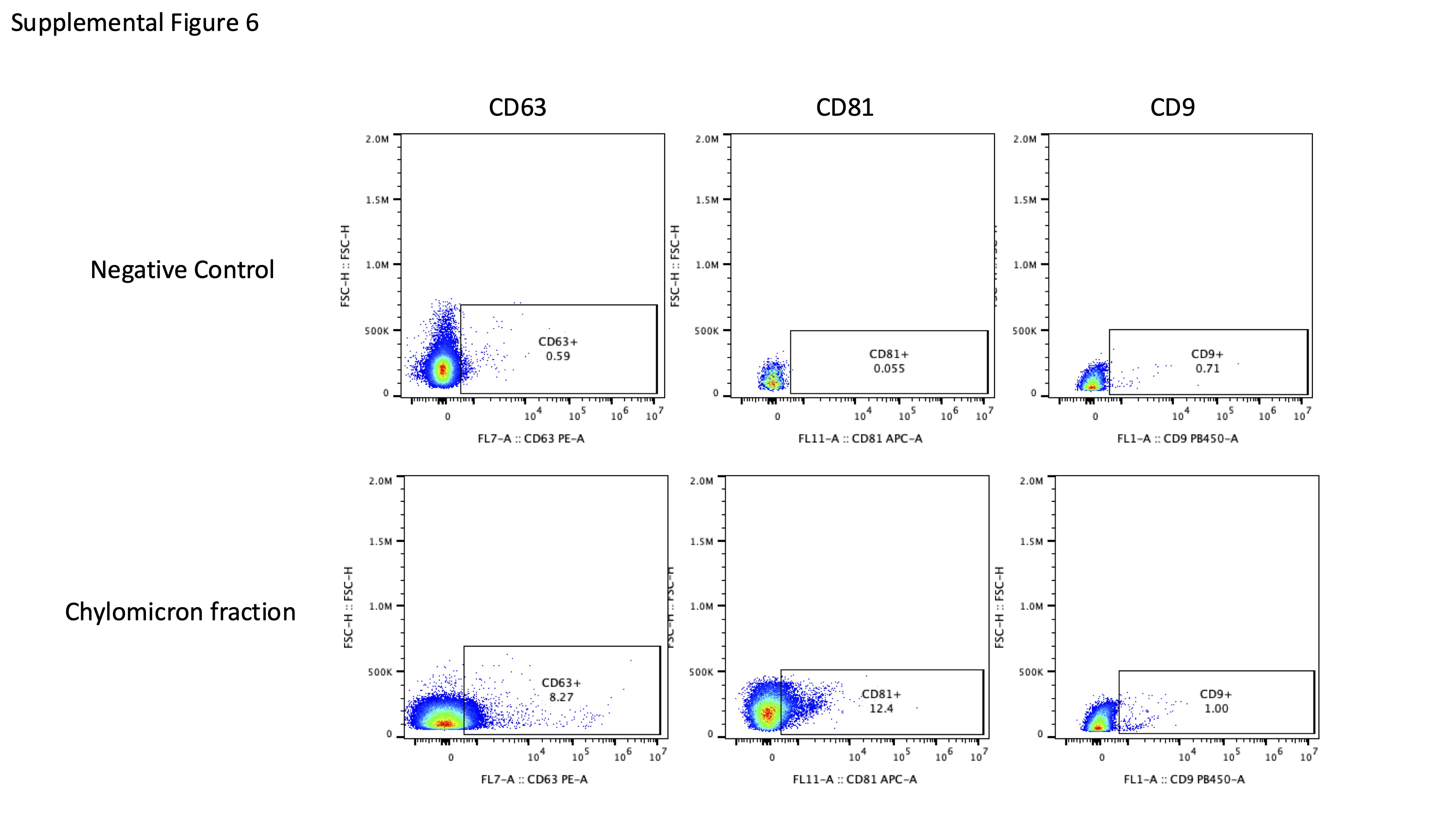

Supplement: Supplementary file 8 — Supporting Figure 6: CD63, CD81, CD9 and ApoB expression in chylomicron fraction. Representative pseudocolor plots of CD63, CD81 and CD9 expression in negative control and in chylomicron fractions. [file JEX2-5-e70170-s012.jpg]

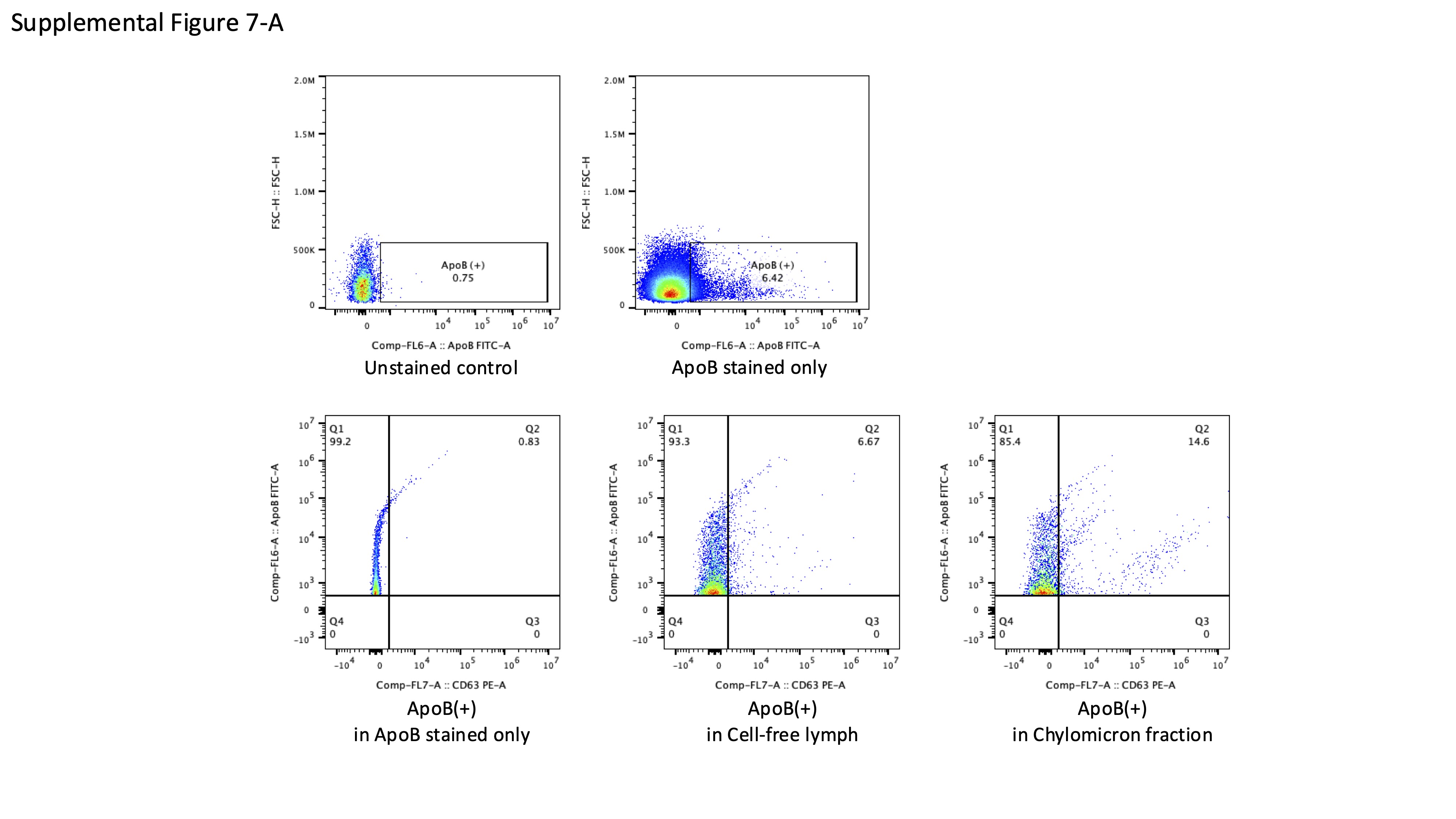

Supplement: Supplementary file 9 — Supporting Figure 7A: CD63, CD81, CD9 expression on ApoB+ particles in chylomicron fraction. Representative pseudocolor plots of CD63 (A), CD81 (B) and CD9 (C) expression, and gating stratergies in negative control (unstained control and ApoB stained only sample), cell‐free lymph collected 2 h after lipid infusion, and in chylomicron fractions within ApoB positive population. (D) Representative gating strategies screen shot when using FlowJo for negative control (unstained control and ApoB stained only sample). [file JEX2-5-e70170-s004.jpg]

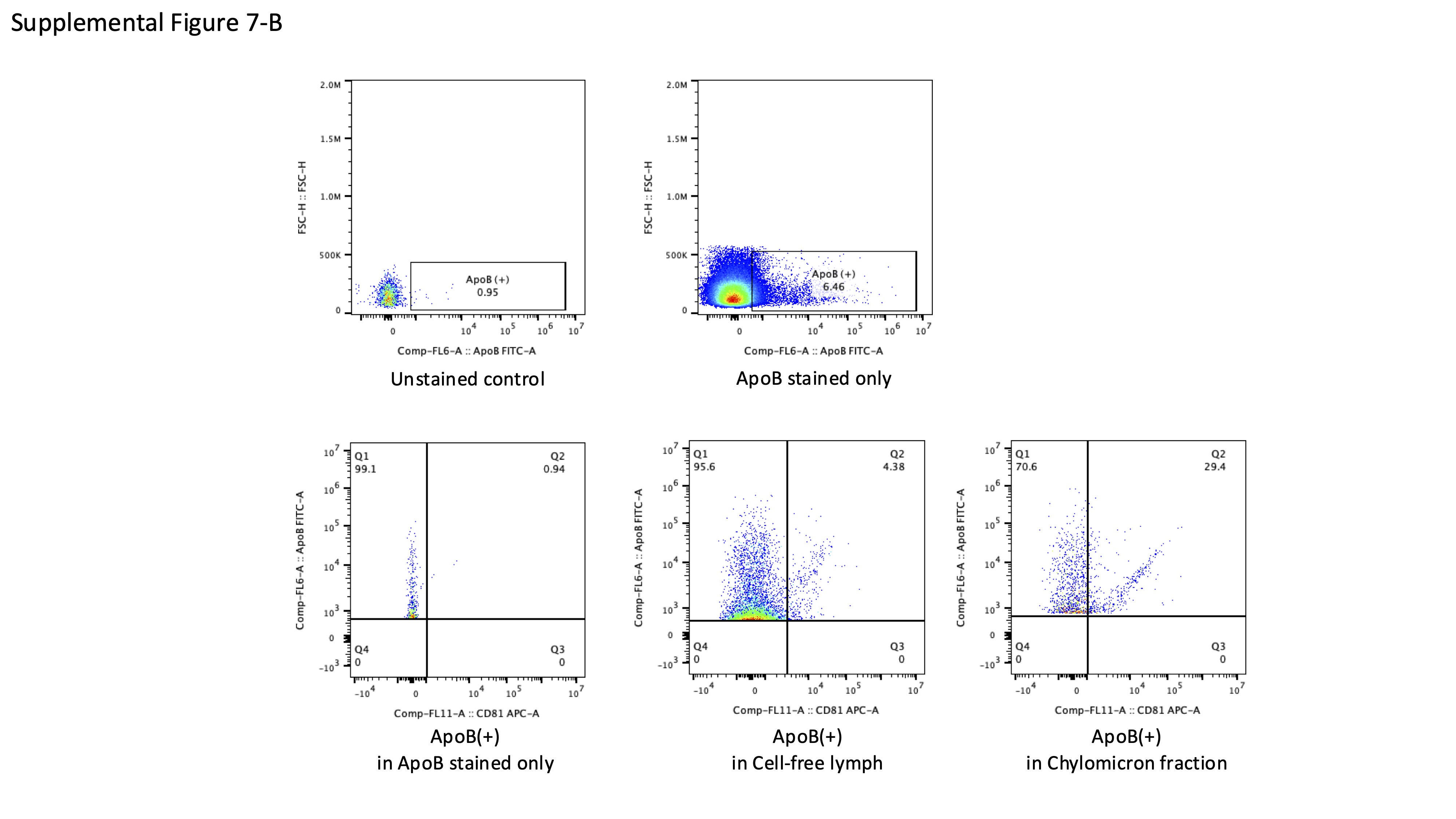

Supplement: Supplementary file 10 — Supporting Figure 7B [file JEX2-5-e70170-s011.jpg]

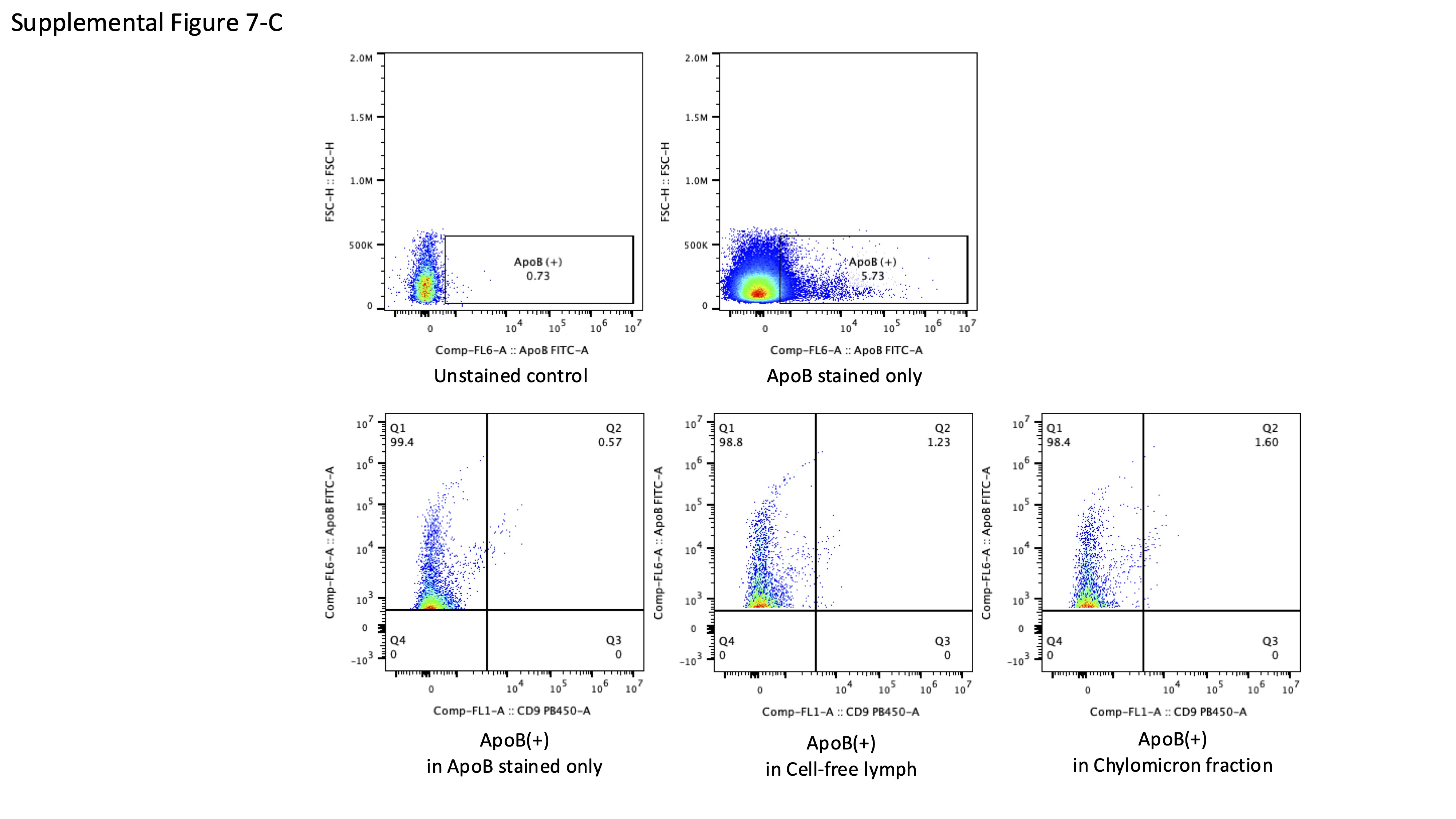

Supplement: Supplementary file 11 — Supporting Figure 7C [file JEX2-5-e70170-s005.jpg]

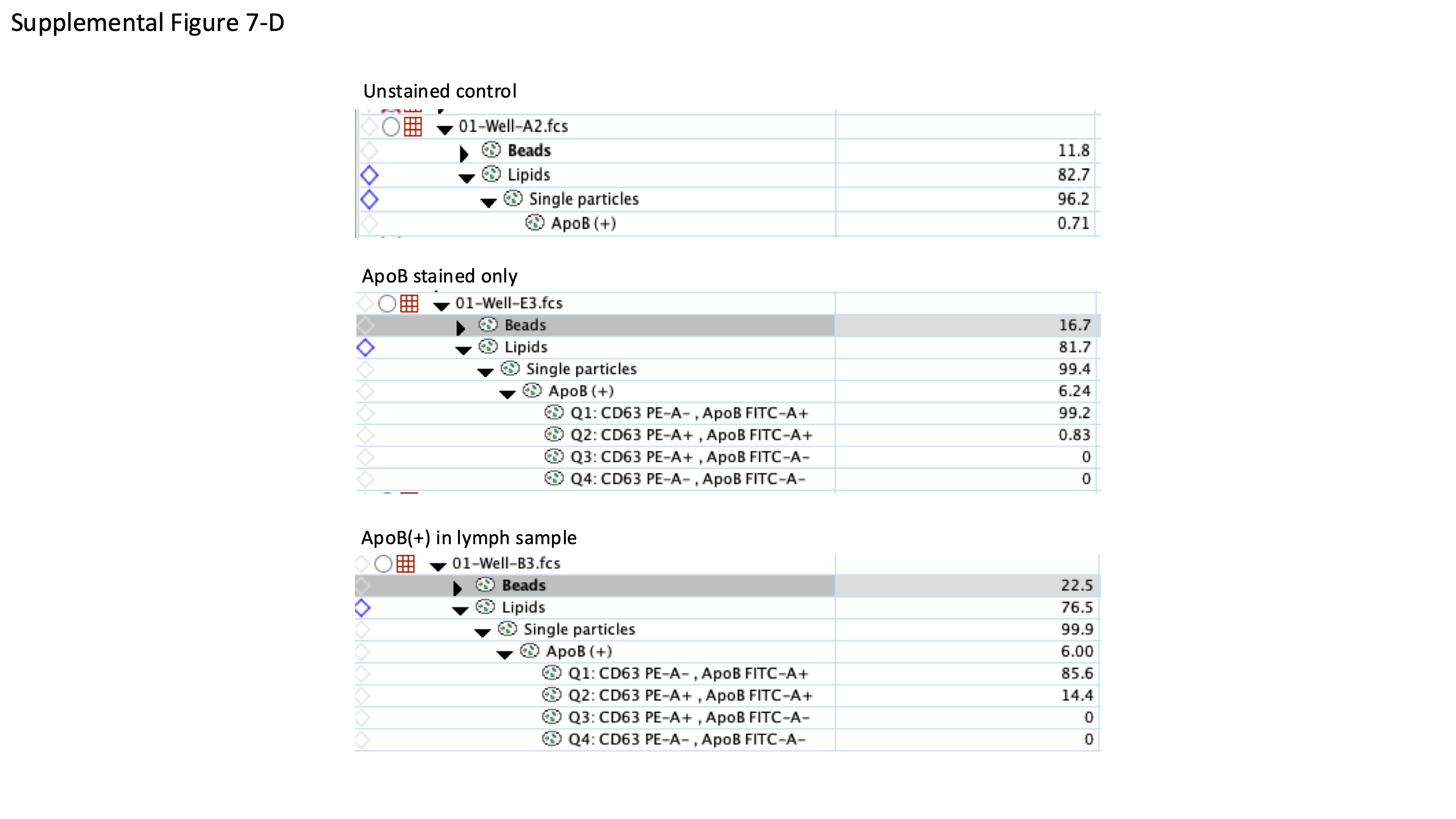

Supplement: Supplementary file 12 — Supporting Figure 7D [file JEX2-5-e70170-s007.jpg]
